# Single xenotransplant of rat brown adipose tissue prolonged the ovarian lifespan of aging mice by improving follicle survival

**DOI:** 10.1111/acel.13024

**Published:** 2019-08-06

**Authors:** Liang‐Jian Chen, Zhi‐Xia Yang, Yang Wang, Lei Du, Yan‐Ru Li, Na‐Na Zhang, Wen‐Yi Gao, Rui‐Rui Peng, Feng‐Yu Zhu, Li‐Li Wang, Cong‐Rong Li, Jian‐Min Li, Fu‐Qiang Wang, Qing‐Yuan Sun, Dong Zhang

**Affiliations:** ^1^ State Key Lab of Reproductive Medicine Nanjing Medical University Nanjing China; ^2^ Department of Center for Medical Experiments Third Xiang‐Ya Hospital of Central South University Changsha China; ^3^ Animal Core Facility Nanjing Medical University Nanjing China; ^4^ Analysis & Test Center Nanjing Medical University Nanjing China; ^5^ State Key Lab of Stem Cell and Reproductive Biology, Institute of Zoology Chinese Academy of Sciences Beijing China

**Keywords:** aging, brown adipose tissue (BAT), lifespan, mice, ovary, rat, xenotransplant

## Abstract

Prolonging the ovarian lifespan is attractive and challenging. An optimal clinical strategy must be safe, long‐acting, simple, and economical. Allotransplantation of brown adipose tissue (BAT), which is most abundant and robust in infants, has been utilized to treat various mouse models of human disease. Could we use BAT to prolong the ovarian lifespan of aging mice? Could we try BAT xenotransplantation to alleviate the clinical need for allogeneic BAT due to the lack of voluntary infant donors? In the current study, we found that a single rat‐to‐mouse (RTM) BAT xenotransplantation did not cause systemic immune rejection but did significantly increase the fertility of mice and was effective for more than 5 months (equivalent to 10 years in humans). Next, we did a series of analysis including follicle counting; AMH level; estrous cycle; mTOR activity; GDF9, BMP15, LHR, Sirt1, and Cyp19a level; ROS and annexin V level; IL6 and adiponectin level; biochemical blood indices; body temperature; transcriptome; and DNA methylation studies. From these, we proposed that rat BAT xenotransplantation rescued multiple indices indicative of follicle and oocyte quality; rat BAT also improved the metabolism and general health of the aging mice; and transcriptional and epigenetic (DNA methylation) improvement in F0 mice could benefit F1 mice; and multiple KEGG pathways and GO classified biological processes the differentially expressed genes (DEGs) or differentially methylated regions (DMRs) involved were identical between F0 and F1. This study could be a helpful reference for clinical BAT xenotransplantation from close human relatives to the woman.

## Graphical abstract

Prolonging ovarian lifespan is attracting and challenging. Herein, we found that one-time rat-to-mouse BAT xenotransplantation did not cause serious global rejection reaction, while significantly prolonged the ovarian lifespan and improved the overall health condition of aging mice. Mechanical studies revealed that the number of antral follicles, which will conduce to live birth, increased onefold through a combined improvement at epigenetic, transcriptional, and protein level.

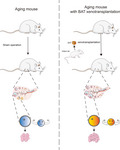

**Abbreviations**
ALT: glutamic pyruvic aminotransferase
AST: glutamic oxalacetic aminopherase
BAT: brown adipose tissue
CK: creatine kinase
Fkkbp6: FK506 binding protein 6
GLU: glucose
GV: germinal vesicle
HLA‐A: major histocompatibility complex, class I, A
MCAD: acyl‐Coenzyme A dehydrogenase, medium chain
MII: second metaphase
MTM: mouse‐to‐mouse
PGC1α: peroxisome proliferative activated receptor, gamma, coactivator 1 alpha
PPARα: peroxisome proliferator‐activated receptor alpha
RNA‐seq: RNA sequencing
ROS: reactive oxygen species:
RRBS: reduced representation bisulfite sequencing
RTM: rat‐to‐mouse
Sall4: spalt‐like transcription factor 4
UA: uric acid
UCP‐1: uncoupling protein 1

## 1 INTRODUCTION

The mechanism of aging is a hot topic in medical studies since many people dream of living a long and healthy life. The lifespan of the ovaries, which produce fertile oocytes and sex hormones, is much shorter than the total lifespan due to the continuous atresia of nonrenewable follicles. Many studies aim at resolving how different primordial follicles are guided toward different destinies: rest, develop, or move toward atresia (Adhikari et al., 2009; Reddy, Zheng, & Liu, 2010). Among the involved mechanisms, the MTORC1 signal pathway has been shown to be a critical regulatory system. In the ovaries, regular mTOR activity is essential for follicle survival, activation, and growth. Normally, mTOR activity is regulated by two upstream branches: PI3K‐Akt and LKB1‐AMPK (Chen et al., 2013; Guo et al., 2018; Hsueh, 2014). The depletion of critical components of the mTOR complex (Guo et al., 2018) or those of its upstream positive regulators, such as Fox3a (Castrillon, Miao, Kollipara, Horner, & DePinho, 2003) or PDK1 (Reddy et al., 2009), causes infertility due to a lack of mature follicles; knockout of its inhibitory regulators, such as PTEN (Reddy et al., 2008) or LKB1 (Jiang et al., 2016), causes excessive follicle activation, premature ovarian insufficiency, and oocyte apoptosis.

Based on the mechanistic studies above, mTOR inhibitors were used to lessen the pathological or chemotherapy‐induced overactivation of follicles or to partially inhibit follicular activation to prolong the time of follicular depletion during natural aging (Dou et al., 2017; Goldman et al., 2017; Li et al., 2015; Shay et al., 2017; Smith et al., 2013; Zhou et al., 2014). For example, the direct mTOR inhibitor rapamycin has been shown to prevent chemotherapy‐induced hyperactivation of the follicles while maintaining the primordial follicle pool (Dou et al., 2017; Goldman et al., 2017).

In addition to the mTOR signal, various adverse factors that could induce a high level of inflammation, reactive oxygen species (ROS), DNA damage, and molecules directly linked with the apoptosis pathway might induce apoptosis and affect ovarian follicle survival. A reduction in these adverse factors could potentially improve follicle quality and extend the ovarian lifespan (Liew et al., 2014; Shen et al., 2017; Uri‐Belapolsky et al., 2014). For example, the mRNA and protein levels of the proinflammatory cytokine IL‐1α are enriched within developing follicles (oocytes and granulosa cells), and their ovarian mRNA levels increase with age. IL‐1α knockout significantly increased female fertility in 12‐month‐old mice, and the number of developing follicles (secondary and antral), the anti‐Mullerian hormone (AMH) levels, and the FSHR levels were all considerably elevated (Uri‐Belapolsky et al., 2014). Several other compounds, such as anethole (Sa et al., 2018), erythropoietin (Mahmoodi, Soleimani Mehranjani, Shariatzadeh, Eimani, & Shahverdi, 2014), N‐acetylcysteine (Mahmoodi, Soleimani Mehranjani, Shariatzadeh, Eimani, & Shahverdi, 2015), and melatonin (Hemadi et al., 2009), were reported to function as antioxidants and promote follicle survival.

In addition, adult stem cells and tissue xenografts have been employed to treat premature ovarian failure and prevent natural ovarian aging (Ding et al., 2018; Gao et al., 2013). For example, human adipose‐derived stem cells (ADSCs) transplanted into the ovaries of naturally aged mice significantly increased the number of follicles at all developmental stages. A protein microarray analysis showed that the levels of basic fibroblast growth factor (bFGF) and hepatocyte growth factor (HGF) were markedly higher than those of other factors, and a mechanistic study has demonstrated that bFGF and HGF reduce ROS levels by upregulating SIRT1 (Ding et al., 2018).

Although researchers have developed programs to slow follicle activation and improve follicle quality to prolong ovarian life, these programs have many shortcomings as mentioned above. First, none of these activators or inhibitors specifically targets the ovaries, so they might affect other organs as well. Second, these activators or inhibitors might cause side effects; for example, the mTOR inhibitor rapamycin is an immunosuppressant that could lead to adverse metabolic reactions (Dou et al., 2017; Goldman et al., 2017; Zhou et al., 2017). Third, although adult stem cells have been shown to be active and have a low toxicity, they have to be transplanted into the ovary (Ding et al., 2018; Gao et al., 2013), which could not be effective for the long term and could induce tumorigenesis.

In several studies, it was recently proven that the simple subcutaneous transplantation of brown adipose tissue (BAT) into the scapular region could treat obesity and metabolic diseases, such as type 2 diabetes mellitus and dyslipidemia (Ding et al., 2018; Villarroya, Cereijo, Villarroya, & Giralt, 2017; Villarroya, Cereijo, & Villarroya, 2013; Wang, Zhao, & Lin, 2015). Recently, the pregnancy and delivery rates in mice were successfully recovered after rat‐to‐rat (RTR) BAT allotransplantation, but a long‐term, continuous fertility assay was not conducted (Yuan et al., 2016). However, if this strategy is applied in future clinical trials, it will be difficult or expensive to obtain BAT from a healthy, young donor woman.

Therefore, in our attempts to increase female fertility, we posed several questions: Could BAT transplantation improve ovarian function and prolong ovarian lifespan in naturally aging mice? Could we use BAT from another species to permanently resolve the BAT donor problem? Would cross‐species transplantation cause harmful xenograft rejection to occur? To investigate these questions, we took advantage of the common rat‐to‐mouse (RTM) xenograft model and performed rat BAT transplantation into 8‐month‐old aging mice concurrently with an MTM group. We found that RTM BAT transplantation did not cause obvious xenograft rejection and could significantly improve ovarian performance for more than 5 months. We also made interesting discoveries about the underlying mechanism at multiple molecular levels.

## 2 MATERIALS AND METHODS

### 2.1 Animals and experiment grouping

All experiments requiring the use of animals were approved by the Committee on the Ethics of Animal Experiments of Nanjing Medical University. All animals were kept in Animal Core Facility of Nanjing Medical University (ACFNMU, Nanjing, Jiangsu, China).

Four groups were set up in parallel for all the experiments: aging group, aging mice (8‐month‐old retiring mice) accepting sham operation; MTM group, aging mouse receiving allotransplanted mouse BAT; RTM group, aging mouse receiving xenotransplanted rate BAT; and young group, 2‐month‐old young mice receiving sham operation. All aging mice were randomly allocated into three groups (aging, RTM, or MTM). The mice were maintained on a 12‐hr light: 12‐hr darkness cycle and were provided with food and water ad arbitrium.

For initial verification of the survival of the xenotransplanted BAT, two rat strains, SD‐Tg(CAG‐LSL‐tdtomato)16/Nju (tdtomato, Cat No: T004476) and SD‐H11‐CNPase‐Cre Cas9‐KI (H11‐CNPase‐Cre, Cat No: T002166), were introduced from Model Animal Research Center of Nanjing University (Nanjing, China). Tomato‐expressing F1 rats were obtained by crossing female tdtomato F0 rat with male H11‐CNPase‐cre F0 rat and PCR verification of the presence of both tdtomato and H11‐CNPase‐cre. The results F1 rat will have global tomato expression (due to H11 promoter) while stronger tomato expression in neuron tissue (due to CNPase promoter). Primers for tdtomato (expected size, 734bp) are as follows: forward (TdTomato‐tF1), 5′‐CCTCCTCCGAGGACAACAAC‐3′; reverse (3108‐pA‐tR1), 5′‐ATAGGCAGCCTGCACCTGAG‐3′. Primers for H11‐CNPase‐Cre (expected size, 414bp) are as follows: forward (Rat‐H11‐tF1), 5′‐ATAATCCTTCAGCTGCCCAGTCTGC‐3′; reverse (CNPase‐tR1), 5′‐CAGATCCACTAGAAGAGGGCATCAGA‐3′.

For mating experiments, CD1 adult male mice (10–12 weeks of age) were purchased from ACFNMU. One mating cage contained one female and one male. To avoid any probable effects caused by the behavior change of male mice, all male mice rotated among different female cages according to a random‐rotating table (Dataset S1).

For other experiments, mice were group‐housed at up to five mice per cage.

For subsequent BAT donor, 2‐week‐old female CD1 mice or SD rats were also purchased from ACFNMU.

### 2.2 BAT transplantation

BAT transplantation was conducted in sterile conditions according to the methods described previously (Yuan et al., 2016). Two‐week‐old female CD1 mice or SD rats were anesthetized with avertin (400 mg/kg body weight i.p.), and the BAT around the scapula was taken out and placed in sterile saline. In the meanwhile, the recipient rats/mice were anesthetized, and then, the donor BAT was transplanted into the scapular region of the recipients. Mice from young or aging group accepted sham operation: The skin covering the scapula region was cut and then stitched without BAT transplantation. All the postoperative mice recovered for 3–4 weeks for the wound healing. Then, the mice were used for mating assay or all other experiments.

### 2.3 Antibodies

Mouse monoclonal FITC‐conjugated anti‐a‐tubulin antibodies (Cat#: F2168) and mouse monoclonal anti‐b‐actin antibodies (Cat#: A5441) were purchased from Sigma; mouse monoclonal GAPDH antibody was purchased from Beyotime (Cat#:AF0006); and RPS6 (phospho‐S240) polyclonal antibody, AMPKα1/2 (phospho‐T183/172) polyclonal antibody, and UCP1 polyclonal antibody were purchased from Bioworld (Cat#: BS4359, BS4457 and BS70689). Rabbit polyclonal anti‐HLA antibody was purchased from Thermo (Cat#:MA5‐11723). Rabbit polyclonal anti‐adiponectin antibody and rabbit anti‐Il6 antibody were purchased from Affinity Biosciences (Cat#:DF7000 and DF6087).

### 2.4 Immunoprecipitation and LC‐MS identification

Five micrograms of rabbit anti‐UCP1 antibody was first coupled to 30 μl protein A/G beads (Macgene) for 4 hr at 4°C on a rotating wheel in 250 μl IP buffer (20 mM Tris‐HCl, pH 8.0, 10 mM EDTA, 1 mM EGTA, 150 mM NaCl, 0.05% Triton X‐100, 0.05% NP‐40, 1 mM phenylmethylsulfonyl fluoride) with 1:100 protease inhibitor (Sigma) and 1:500 phosphatase inhibitor (Sigma). Meanwhile, 10 mg BAT at the transplantation region was lysed and ultra‐sonicated in 250 µl IP buffer and then precleaned with 30 µl protein A/G beads for 4 hr at 4°C. Then, protein A/G‐coupled UCP1 antibody was incubated overnight at 4°C with precleaned BAT lysate supernatant. Finally, after three washes (10 min each with 250 ul IP buffer), the beads with bound immune complexes were subjected to Western blotting SDS‐PAGE and silver staining, and the gel strip at UCP1 position (MW:33 kDa) was cut out and sent to Shanghai Bioprofile Inc. for UCP1 protein identification (rat UCP1 or mouse UCP1) through LC‐MS.

### 2.5 Paraffin section preparation and Immunohistochemistry

Brown adipose tissues were fixed in 10% buffered formalin for paraffin embedding and sectioning. After deparaffinization and rehydration, sections were processed for blocking of endogenous peroxidase activity and antigen retrieval pretreatment. Immunohistochemical analyses were performed using a SPlink Detection Kits (Zhong Shan Jin Qiao) with antibodies against Ucp1 overnight at 4°C. Negative controls were treated by incubation with nonimmune rabbit IgG.

For BAT tomato fluorescence image taking, the paraffin section was stained with 1 µg/ml DAPI for 20 min, and then, the sample was observed, and confocal images were taken under an Andor Revolution spinning disk confocal workstation.

### 2.6 Real‐time RT–PCR

Total RNA was isolated from ovaries in each group by RNAprep pure Tissue Kit (TIANGEN Biotech) according to the manufacturer's instructions and was quantified with a spectrophotometer (NanoDrop 2000c, Thermo Fisher Scientific). RNA (500 ng/reaction per sample) was reverse‐transcribed using a FastQuant RT Kit (TIANGEN Biotech) to create cDNA. Quantitative real‐time PCR then was performed using Eva Green qPCR Master mix (Applied Biological Materials Inc.) on an ABI Step One Plus platform (Thermo Fisher Scientific). The specificity of the PCR products was assessed by melting curve analyses, and amplicon size was determined by electrophoresis in 2% agarose gels. Quantification of various mRNAs was performed by using the actin amplification signal as an internal control. The specific primers used are shown in Table S1.

### 2.7 Ovarian follicle counting

Ovaries were collected and fixed in 10% buffered formalin for 12 hr, embedded in paraffin, serially sectioned at a thickness of 5 µm, and then stained with hematoxylin and eosin. All follicles with a visible nucleus were counted every second section. Follicle classification was determined by Pederson's standard: Oocytes surrounded by a single layer of flattened or cuboidal granulosa cells were defined as primordial or primary follicles; oocytes surrounded by more than one layer of cuboidal granulosa cells with no visible antrum were determined to be secondary follicles. Antral follicle possessed a clearly defined antral space and a cumulus granulosa cell layer. Corpora lutea were filled with lutein cells, and follicles were considered atretic if they contained either a degenerating oocyte, disorganized granulosa cells, pyknotic nuclei, shrunken granulosa cells, or apoptotic bodies. The results were reported as the number of follicles counted per ovary.

### 2.8 Estrous cycle analysis

Vaginal smears were collected on glass slides in 10 µl of 0.9% NaCl at 07:00–08:00 every morning. After air‐drying, samples were stained with Toluidine Blue O (Amresco) for 3–4 min, and then washed and dried. The four stages of the estrous cycle were determined as previously described by analyzing the proportion of three major cell types (epithelial cells, cornified cells, and leukocytes). Consistent cycles of proestrus, estrus, metestrus, and diestrus (4–5 days total) in mice were called “regular estrous cycle.”

### 2.9 Oocyte collection and culture

Immature oocytes detained in prophase I, that is, germinal vesicle (GV) oocytes, were obtained from the ovaries of 3‐ to 4‐wk‐old ICR female mice. Mice were first anesthetized with CO_2_, then were euthanized by cervical dislocation, and ovaries were isolated and placed in operation medium (Hepes) with 2.5 nM milrinone and 10% fetal bovine serum (FBS; Thermo Fisher Scientific). Oocytes were dismissed from the ovary by puncturing follicles with a hypodermic needle. Cumulus cells were washed off cumulus–oocyte complexes, and every 50 isolated denuded oocytes were placed in 100 ml droplets of culture medium under mineral oil (MilliporeSigma) in plastic dishes (BD). Culture medium used was minimum essential medium (MEM) supplemented with 0.01 mM EDTA, 0.23 mM sodium pyruvate (Sigma), 0.2 mM penicillin/streptomycin (Gibco), and 3 mg/ml bovine serum albumin (Sigma) that contained 20% FBS (Gibco). Oocytes were grown at 37°C, 5% O_2_, and 5% CO_2_ in a humidified atmosphere. Before in vitro maturation, all culture media included 2.5 nM milrinone to prevent the resumption of oocyte meiosis.

### 2.10 Measurement of ROS levels

Intracellular ROS levels were determined by CM‐H2DCFDA (Cat#: S0033; Beyotime Biotechnology TM). CM‐H2DCFDA was prepared in DMSO prior to loading. Oocytes were incubated in M16 medium containing 5 mM CM‐H2DCFDA for 20 min at 37°C in a 5% CO_2_ incubator. After three washes, 20 oocytes were loaded on a slide with a microdrop of medium and immediately observed under a Laser Scanning Confocal Microscope (LSM 710, Zeiss).

### 2.11 Assay of mitochondrial membrane potential

Oocytes were incubated at 37°C for 20 min with JC‐1 diluted at 1:200 (40706ES60, YEASEN), then washed twice with PBS and placed in 50ul droplets of culture medium. A green fluorescence (JC‐1 as a monomer at low membrane potentials) and a red fluorescent (JC‐1 as “J‐aggregates” at higher membrane potentials) were captured as above. Mitochondrial depolarization is indicated by a decrease in the red/green fluorescence intensity ratio.

### 2.12 Measurement of body temperature through infrared thermography

Mice were exposed to a cold chamber (4°C) with one mice per cage for up to 4 hr, with free access to food and water. Images were taken using a ONE PRO Compact Infrared Thermal Imaging Camera (FLIR). Body temperature was measured using an infrared thermometer (Xiaomi ihealth Instruments). The thermometer was always kept 1 cm above the back.

### 2.13 RNA sequencing and analysis

RNA samples were collected from mouse ovaries. RNA isolation, library construction, and RNA sequencing (RNA‐seq) were carried out by the Beijing Genomics Institute following standard protocols. The library products were sequenced using a BGISEQ‐500. Standard bioinformatics analysis was performed by the Beijing Genomics Institute. For gene expression analysis, the significance of the differential expression genes was defined by the bioinformatics service of BGI according to the combination of the absolute value of |log2 Ratio| ≥ 1 and *q* value < 0.001. All original sequence datasets have been submitted to the database of NCBI Sequence Read Archive (SRA) under accession number.

### 2.14 Reduced representation bisulfite sequencing (RRBS)

Frozen ovaries were briefly ground in lysis buffer, and then, DNA was extracted with QIAamp DNA Blood Maxi Kit (Qiagen) according to the manufacturer's recommendation. Reduced representation bisulfite sequencing was performed by Shanghai BioGenius Biotech Inc as previously described (Meissner et al., 2005) (31). In short, DNA samples were bisulfite‐treated with EZ DNA Methylation‐Gold kit (ZYMO Research) to create the library. Sequencing reads were converted and loaded on Illumina HiSeq 3000 platform. Differentially methylated regions analysis was performed by swDMR software.

### 2.15 Statistical analysis

All experiments were replicated at least three times, and the data obtained were subjected to statistical analysis. Data are presented as mean ± *SEM*, unless otherwise indicated. Differences between two groups were analyzed by Student's *t* test. Multiple comparisons between more than two groups were analyzed by one‐way ANOVA test using Prism 7.0. *p* < .05 was considered to be significant.

## 3 RESULTS

### 3.1 Xenotransplanted rat BAT was functional and did not cause injurious tissue rejection in aging mice

**Figure 1:**
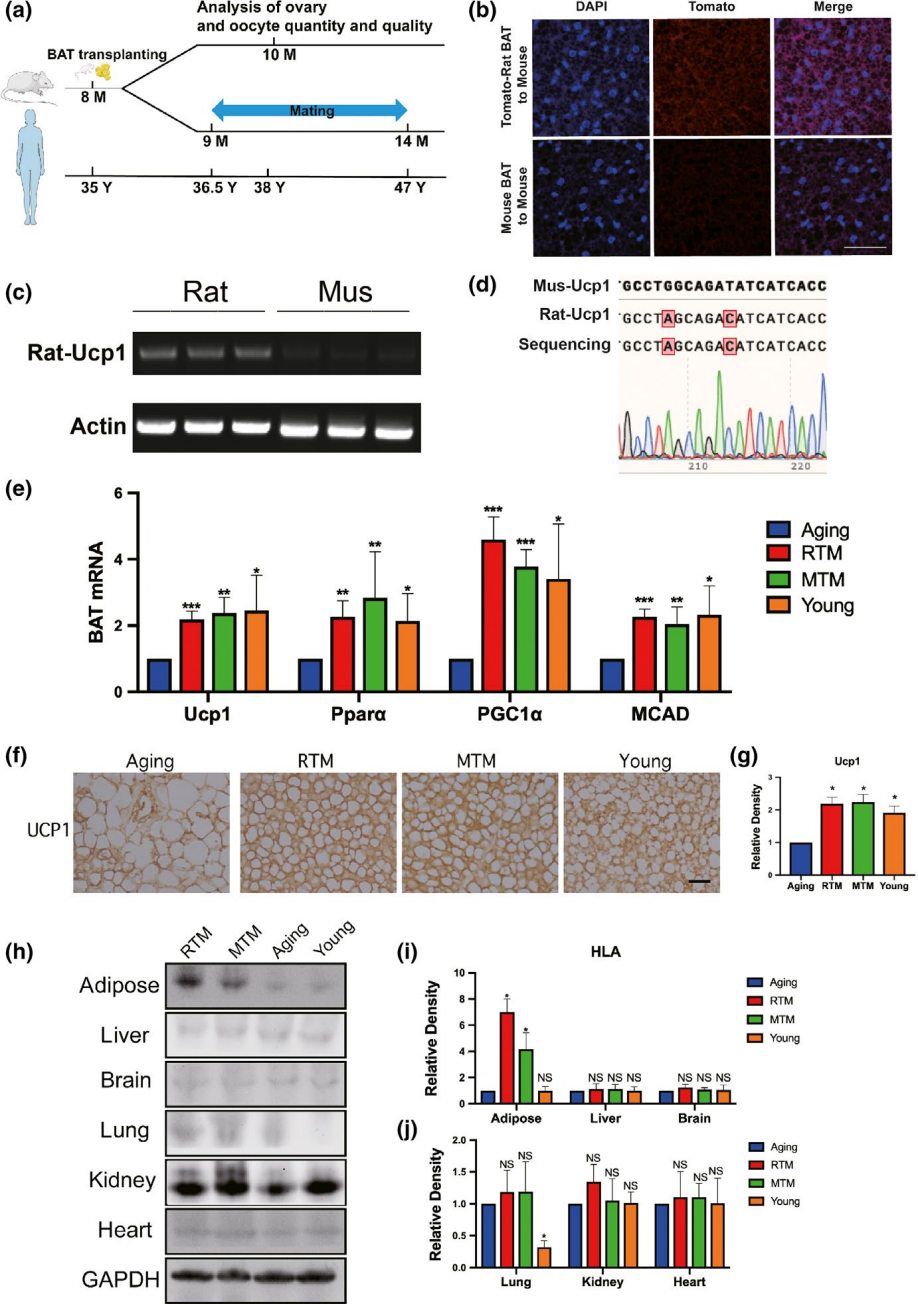
Rat‐to‐mouse (RTM) xenotransplanted brown adipose tissue (BAT) was functional well and did not cause injurious histocompatibility in aging mice. (a) Scheme of experimental design. Eight‐month‐old aging mice were divided into four groups, RTM and MTM groups received brown adipose tissue (BAT) from 1‐month‐old rats or mice, then recovered for 1 month. Aging mice accepted a pseudo operation (cut and stitch up without BAT transplantation). At 9 months old, mating assays started, five mice for each group. At about 10 months old, when the first litter is delivered, more mice were used for all other experiments. The age line of human corresponds to age in mice. (b–d) Verification of xenotransplanted rat BAT 3 weeks after xenotransplantation. (b) BAT paraffin sections were stained with DAPI (blue) and fluorescence image showed that tomato fluorescence (red) was very remarkable in the RTM group while very low in the MTM group. (c) RT–PCR with rat‐specific UCP1 primers detects a bright band with expected size from all three RTM mice, while no observable bands from three MTM mice. (d) The bright band from (c) was identified to be rat UCP1 by Sanger sequencing. (e) qPCR showed that the mRNA levels of four BAT marker genes, UCP1, PPARα, PGC‐1α, and MCAD, were significantly rescued close to the young group. (f) UCP1 immunohistochemistry in BAT paraffin section showed that UCP‐1 level in RTM and MTM group, and young group were significantly higher than in the aging group. (g) Quantification of (f). (h) HLA‐A blot showed that HLA‐A level in BAT of RTM and MTM groups was significantly higher than aging or young group; HLA‐A level in the lung of young group is significantly low; HLA‐A levels in other main tissues (liver, spleen, brain, kidney, heart) are similar among all four groups. (i,j) Quantification of HLA‐A levels in (g). Scale bar, 20 µm. *p < 0.05, **p < 0.01, ***p < 0.001 are considered significantly different

As summarized above, allotransplanted BAT has been used to treat mouse models of diverse human diseases (Ding et al., 2018; Villarroya et al., 2017, 2013; Wang et al., 2015), but nobody has ever investigated whether it can improve the fertility of aging mice with decreasing ovarian function, so we wanted to address this in the current study. Furthermore, if donor BAT, which is the most potent in infants, could be obtained from another species, then the insurmountable BAT donor problem would no longer exist; thus, the possibility of studying BAT xenotransplantation with human clinical trials might significantly increase. Therefore, we decided to test our hypothesis with 8‐month‐old retired CD‐1 mice as experimental acceptors and 2‐week‐old SD rats as BAT donors (Figure 1a). Eight‐month‐old in mice corresponds to 35‐year‐old in woman (Figure 1a). Additionally, as is well known, the fertility of women starts to decrease at 35 years old, and the physiological functions of some organs in humans also start to decrease at the age of 35. Therefore, in this sense, as multiple other researchers have done (Ben‐Meir et al., 2015; Dou et al., 2017; Wilkinson et al., 2012), we simply referred to the 8‐month‐old retired mice as “aging mice.” We set up four groups: (a) 8‐month‐old control aging group (aging); (b) aging mice with xenotransplanted rat BAT (RTM); (c) aging mice with allotransplanted mouse BAT (MTM); and (d) 2‐month‐old mice (young).

As an initial step, to verify that rat BAT is still functional after transplantation, we used a tomato transgenic rat strain and transplanted the BAT around the scapula into the same area in the aging mice. Three weeks after transplantation, the foreign rat BAT in the RTM group began to fuse (a good sign for successful xenotransplantation) with the intrinsic mouse BAT but showed a much stronger fluorescent signal than the signal from the MTM control group around the scapula region (Figure 1b); next, we took out the xenotransplanted rat BAT and verified that it expressed rat‐specific UCP‐1 mRNA, while the mouse BAT (without tomato fluorescence) did not (Figure 1c,d). RT–PCR analysis of BAT marker genes with mouse‐specific primers showed that the mRNA levels of UCP‐1, PPARA, PGC1α, and MCAD in the RTM and MTM groups were all significantly elevated and close to the levels in the young group (Figure 1e). Immunohistochemistry experiments with a UCP‐1 antibody (against rat, mouse, and human; a rat‐specific antibody is not commercially available due to the high similarity been UCP1 of these three species) showed that the RTM and MTM groups showed a UCP‐1 signal significantly stronger than the aging group while close to the young group (Figure 1f,g). Finally, due to the continuous fusion of rat BAT with the mouse BAT, tomato red became undistinguishable after about 3 months. To verify that the rat BAT could function for a long time, we used the UCP1 antibody to perform immunoprecipitation with the BAT lysate at the BAT transplantation region when the mouse was 15 months old (over 6 months after xenotransplantation), and with LC‐MS, we were still able to detect the rat UCP1 protein (Figure S1a, Dataset S2). We also verified the expression of rat UCP1 by PCR and Sanger sequencing (Figure S1b). UCP1 is a well‐known BAT marker protein to regulate heat generation. The results could at least indicate that the xenotransplanted rat BAT could produce functionally important rat BAT‐specific protein even after 6 months of xenotransplantation.

Immune rejection is always a major challenge of xenotransplantation. To determine how serious the immune rejection was in the RTM group, we aligned four groups and detected major histocompatibility complex, class I, A (HLA‐A) protein levels in every main tissue 3 weeks after the BAT transplantation; at this time point, the recipient mice were fully recovered from the surgery, while in the meantime, there might be outbreaks of acute rejection. Therefore, the HLA‐A level might be the highest, and examining the HLA‐A level is the most reflective of histocompatibility during xenotransplantation at this timepoint. We found that in the adipose tissue around the scapula, the RTM and MTM groups had much higher HLA‐A levels than the aging and young groups; the RTM group had the highest HLA‐A level. For all other tissues except for the lungs, all four groups had similar HLA‐A levels, while the young group showed the lowest HLA‐A levels in the lungs (Figure 1h–j). Moreover, the transplanted mice were all very healthy (see below for more specific assays).

These results suggest that BAT xenotransplantation did not induce a global rejection reaction and that the xenotransplanted rat BAT could function for a long time.

### 3.2 RTM transplantation significantly increased the fertility of aging mice by improving ovarian performance

**Figure 2:**
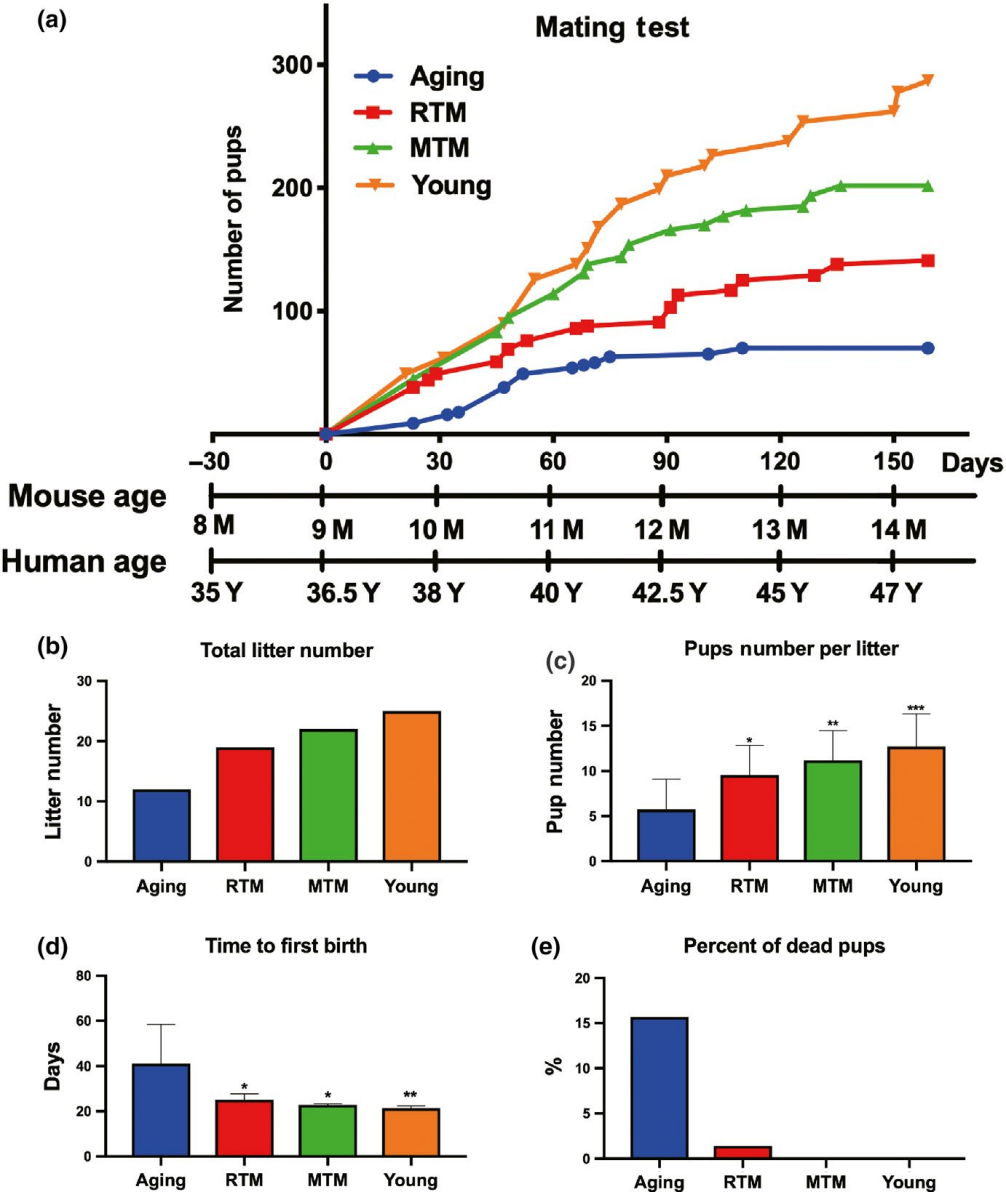
RTM significantly improved the reproductivity of aging mice. (a) Cumulative litter size curve showed that RTM (red curve) significantly increased the fertility of the aging mice. Relative age axes of mouse and human were shown below. (b) Numbers of total litters in RTM, MTM, and young groups were significantly more than in the aging group. (c) Numbers of pups per litter (number of total pup/number of litters) were significantly more in RTM, MTM, and young groups than in the aging group. (d) The time from mating to the first birth was significantly shorter in RTM, MTM, and young groups than in the aging group. (e) Percentage of dead pups in RTM, MTM, and young groups were significantly less than in the aging group. *p < 0.05, **p < 0.01, ***p < 0.001 are considered significantly different

Next, to verify our primary assumption that xenotransplanted rat BAT could improve the fertility of aging mice, we set up five mating cages for each group and started a comparative mating assay for more than 5 months. The results show that compared with the number of total pups from the aging group, the number of total pups from both the RTM and MTM groups significantly increased (Figure 2a, aging:RTM:MTM:young = 70:141:202:287); in addition, the number of litters (Figure 2b, aging:RTM:MTM:young = 12:19:22:25) and the average pup number per litter (number of total pups/number of litters, Figure 2c, aging:RTM:MTM:young = 5.727:9.545:11.181:12.727) also significantly increased in the RTM and MTM groups. Moreover, the time from mating to the first birth (aging/RTM/MTM/young = 41:25:22.8:21.4) (Figure 2d) and the total percentage of dead pups (aging:RTM:MTM:young = 0.157:0.01418:0:0) (Figure 2e) obviously decreased. These results support that xenotransplanted rat BAT could improve the fertility of aging mice.

**Figure 3:**
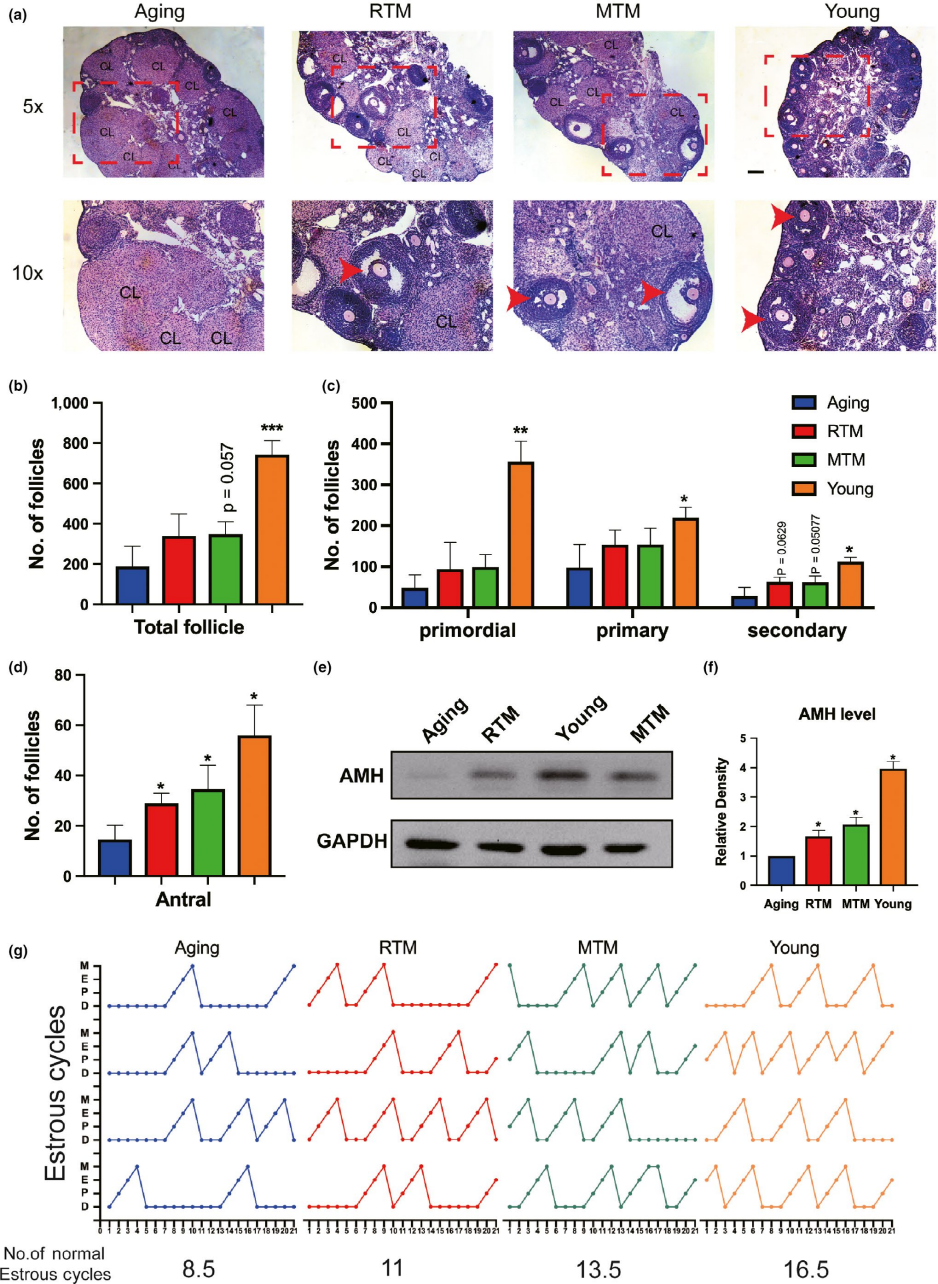
RTM significantly improved the ovarian performance of aging mice. (a–d) Hematoxylin–eosin (HE) staining of continuous ovary paraffin sections (a) and number of ovarian follicles (b–d). (b) for total follicles, (c) for primordial (left), primary (middle), and secondary (right) follicles. (d) for antral follicles. There was no significant difference for the numbers of primordial, primary, secondary, and total follicles between RTM and aging groups, but the number of antral follicles in RTM and MTM groups was a fold more than the aging group. For better vision in (a), a subregion (dot‐line rectangle region) of the upper image (5×) in each group was magnified and put at the lower panel (10×). CL, corps lutein. Antral follicles were arrow‐pointed. (e) Blot showed that AMH levels in RTM, MTM, and young groups were significantly higher than in the aging group. (f) Quantification of AMH levels of ovaries from four groups. (g) Plots of estrus cycle curves within 21 test days, four mice were used for each group. There are more cycles in RTM, MTM, and young groups than in the aging group. Total numbers of estrus cycle from four groups were shown at the bottom of each plot; Scale bar, 200 µm. *p < 0.05, **p < 0.01, ***p < 0.001 are considered significantly different

**Figure 4:**
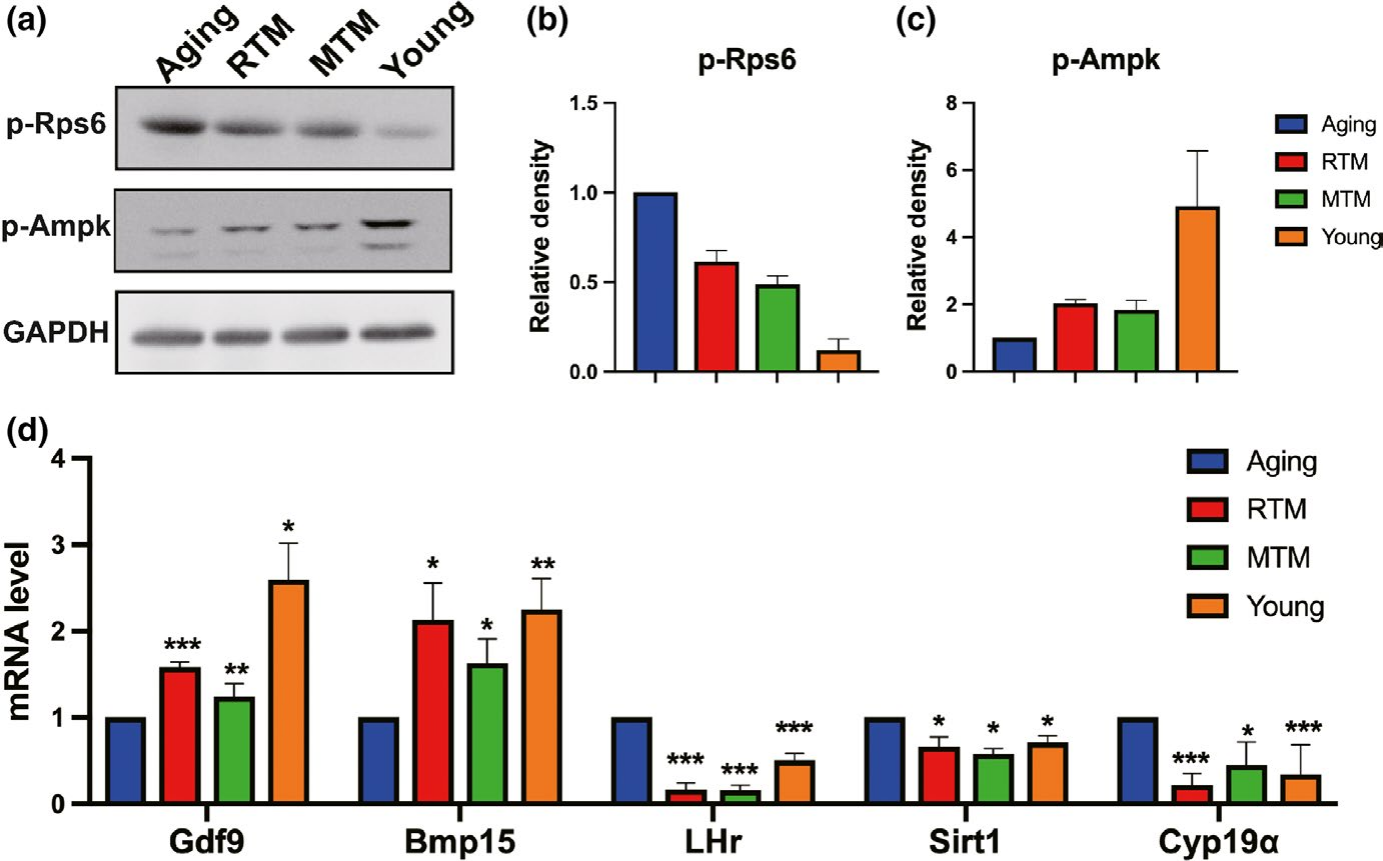
RTM significantly rescued the expression levels of multiple proteins and genes indicative of follicle reserve and oocyte quality. (a) Western blot showed that compared with the aging group, RTM or MTM treatment significantly rescued the protein levels of p‐RPS6 and p‐AMPK close to the young group. (b,c) Quantification of (a). (d) qPCR showed that GDF9 and BMP15 mRNA levels in RTM, MTM, and young groups were significantly higher than in aging group, while mRNA levels of LHR, Cyp19a, and Sirt1 in RTM, MTM, and young groups were significantly lower than in the aging group. *p < 0.05, **p < 0.01, ***p < 0.001 are considered significantly different

Since the fertility in the RTM and MTM groups was significantly improved, we subsequently examined the changes in the ovaries. We found that there were significantly more antral follicles in the RTM and MTM groups than there were in the aging group (Figure 3d). Although there were no statistically significant differences in the number of primordial, primary, and secondary follicles between the RTM, MTM, and aging groups (Figure 3a–c), these numbers tended to increase, and we verified that AMH, a master marker of follicle reserve, significantly increased in the RTM and MTM groups compared with that in the aging groups (Figure 3e,f). Furthermore, there were substantially more normal estrus cycles in the RTM, MTM, and young groups than in the aging group (Figure 4g). These results indicate that xenotransplanted rat BAT improved ovarian reserve and performance.

### 3.3 RTM significantly rescued multiple indices indicative of follicle reserve and oocyte quality in aging mice

From the above results, we deduced that the survival rate and quality of the antral follicles might be improved by BAT transplantation. Next, we tried to find more evidence at the molecular level. First, it is known that proper mTOR activity is essential for follicle reserve, and excessive mTOR activity could cause premature ovarian failure and follicle atresia (Jiang et al., 2016; Reddy et al., 2008, 2009). We found that compared with the aging group, RTM or MTM treatment significantly rescued the protein levels of p‐RPS6 and p‐AMPK to levels that were close to the levels in the young group (Figure 4a–c), indicating that the mTOR signaling pathway was rescued to the proper level. Second, RTM or MTM treatment significantly elevated the mRNA levels of GDF9 and BMP15, two master regulators indicating oocyte quality (Figure 4d). The mRNA levels of LHR and Cyp19a, which are important for normal ovarian steroidogenesis, were returned to the levels of the young group by RTM or MTM treatment (Figure 4d). Moreover, the mRNA level of Sirt1, another essential gene regulating energy homeostasis, apoptosis, and oxidative stress in oocytes, was significantly recovered to a level that was close to that in the young group by RTM or MTM treatment (Figure 4d).

**Figure 5:**
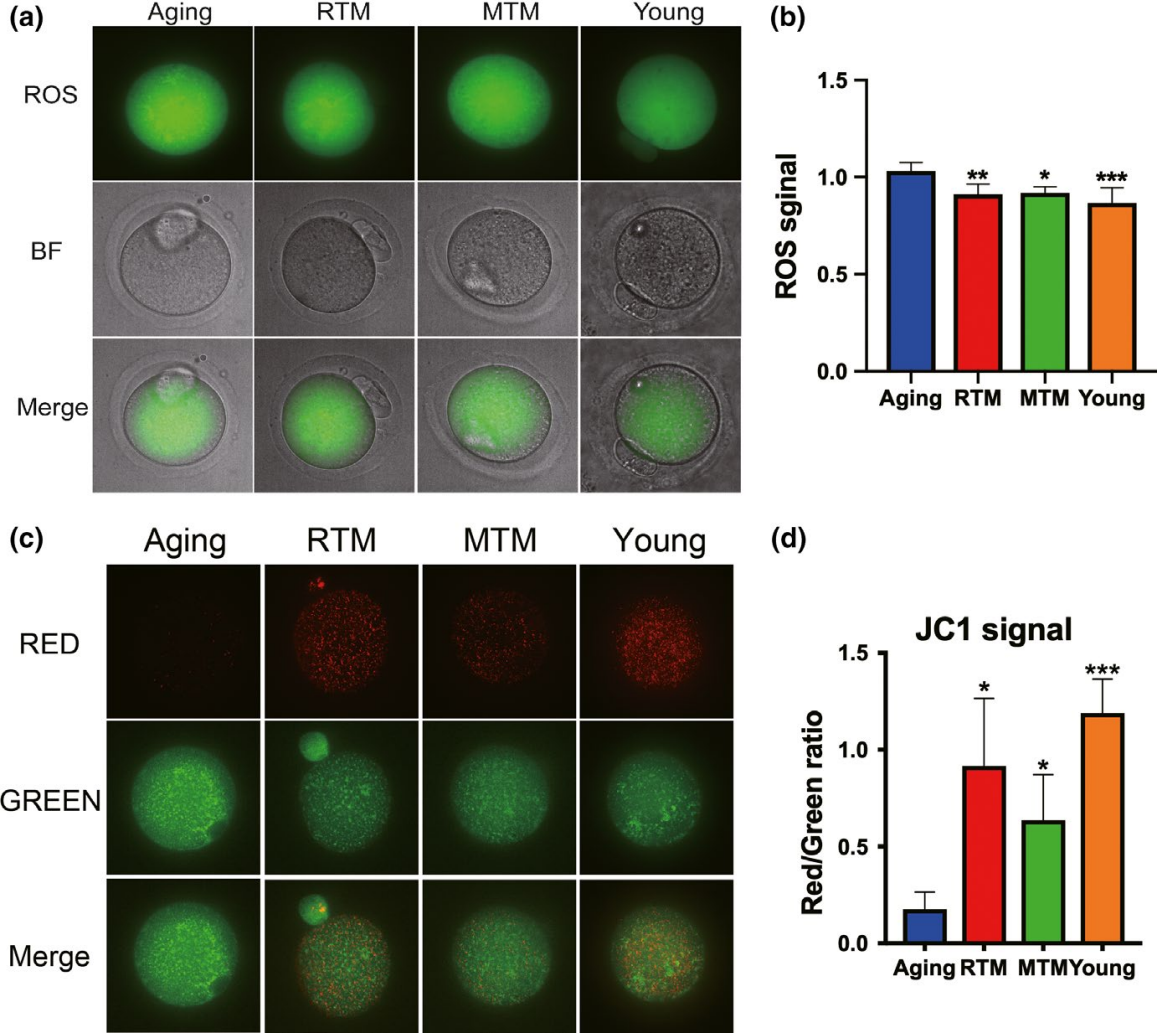
RTM significantly recovered ROS level and membrane potential of aging mice. (a) RTM significantly reduced the reactive oxygen species (ROS) in aging oocytes. (b) Quantification of ROS in (a). (c) RTM significantly elevated the mitochondria membrane potential in aging oocytes. Mitochondria membrane potential is indicated as the fluorescence ratio of red (aggregator)/green (monomer). (d) Quantification of fluorescence ratio in (c). Scale bar, 20 µm. *p < 0.05, **p < 0.01, ***p < 0.001 are considered significantly different

Next, we examined ROS and the membrane potential. Reactive oxygen species are toxic by‐products of aerobic metabolism, and a high level of ROS indicates that aerobic metabolism is abnormally high. Mitochondrial membrane potential is a key marker for mitochondrial integrity. A significantly decreased potential indicates severe dysfunction in mitochondria. We found that the ROS levels in the RTM, MTM, and young groups were significantly lower than those in the aging group (Figure 5a,b). The mitochondrial membrane potentials, as determined by JC‐1 staining, were significantly higher in the RTM, MTM, and young groups than they were in the aging group (Figure 5c,d).

These results indicate that BAT xenotransplantation significantly rescued the levels of multiple markers and indices that indicate follicle reserve and oocyte quality in aging mice.

### 3.4 RTM transplantation significantly improved the metabolism and general health of aging mice

**Figure 6:**
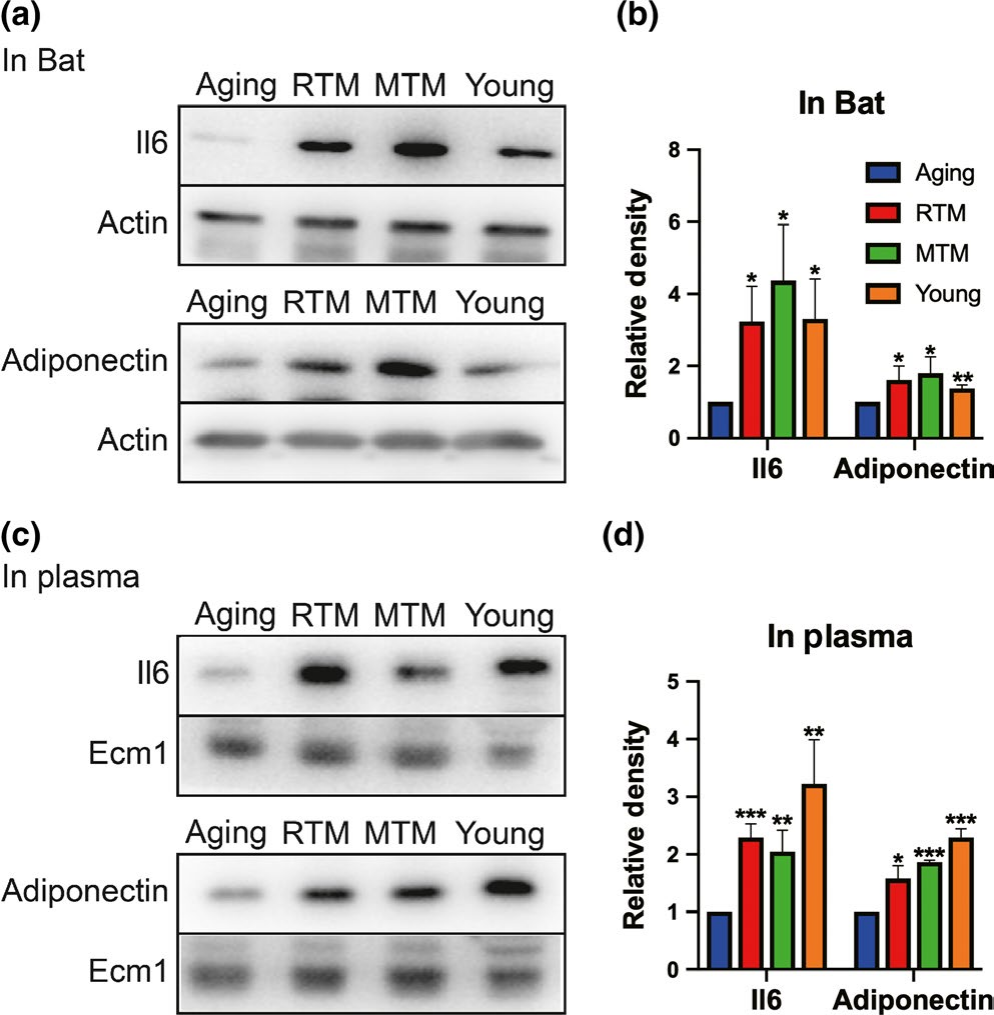
RTM significantly improved the levels of important adipokines of aging mice. (a) The levels of IL6 and adiponectin in BAT were significantly higher in RTM, MTM, and young groups than in the aging group. (b) Quantification of (a). (c) The levels of IL6 and adiponectin in plasma were significantly higher in RTM, MTM, and young groups than in the aging group. (d) Quantification of (c). *p < 0.05, **p < 0.01, ***p < 0.001 are considered significantly different

The fact that BAT xenotransplanted into the scapula region could improve ovary performance indicated that BAT factors (Villarroya et al., 2017, 2013; Wang et al., 2015) could circulate into other tissues to make a positive impact. Next, we examined these adipokines. We found that the levels of IL‐6 and adiponectin, which are known to be important for both thermal and metabolic regulation (Shen, Jiang, Lin, Omary, & Rui, 2019), were significantly elevated in the RTM and MTM groups and were close to levels in the young group in both BAT (Figure 6a,b) and plasma (Figure 6c,d).

**Figure 7:**
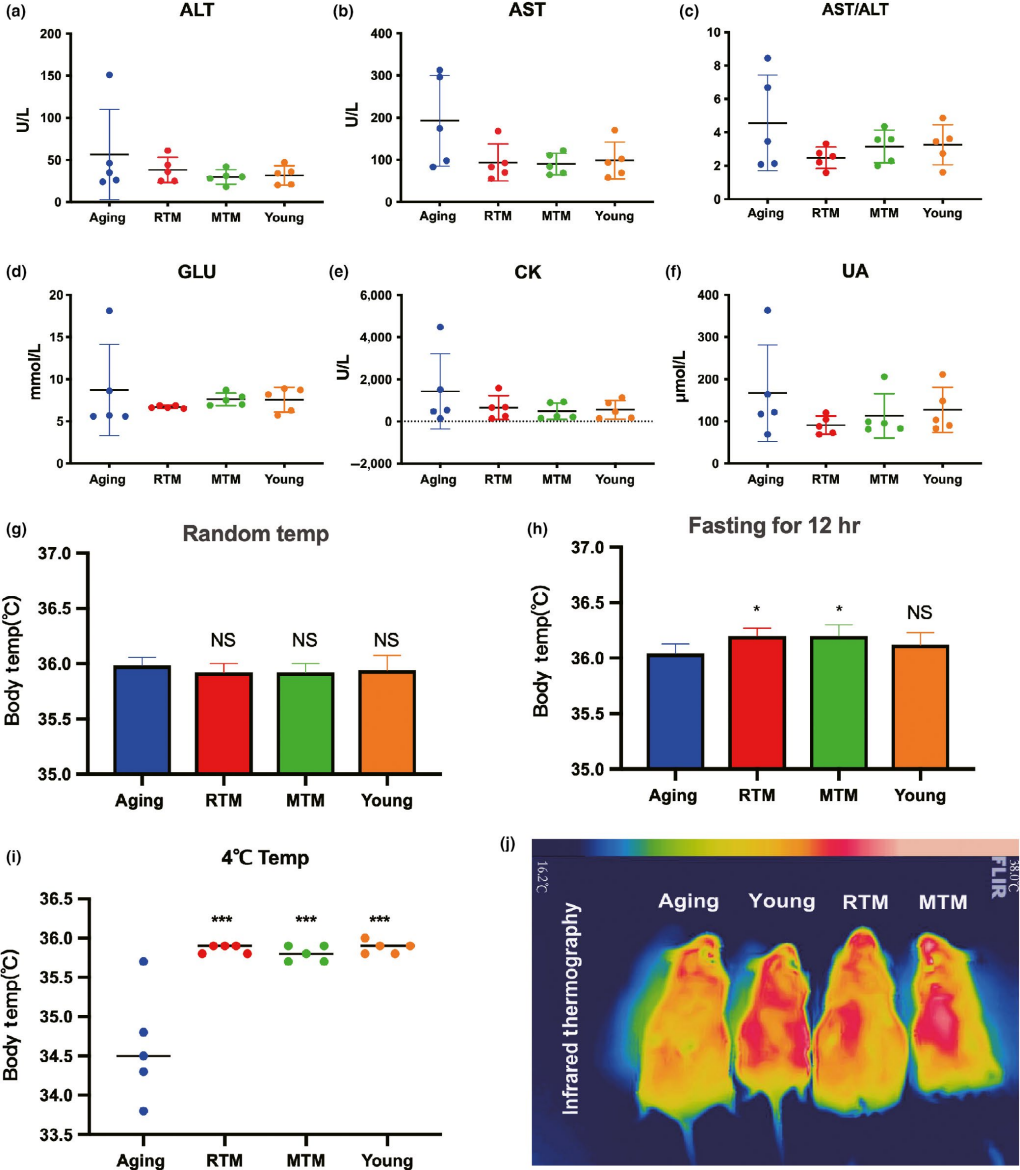
RTM significantly improved the general health of aging mice. (a–f) Measurement of biochemical blood indexes. (a) for AST, (b) for AST, (c) for ALT/AST, (d) for GLU, (e) for CK, and (f) for UA. For each index, five numbers from five different mice (repeats) were plotted. The curves showed that five numbers from RTM, MTM, and young groups were closer to average and distributed within a much narrower region than in the aging group. (g) There were no significant differences between aging, RTM, MTM, and young groups for the body temperature under normal condition. (h) After 12‐hr fasting, the body temperature in RTM or MTM was significantly higher than in the aging group. (i) Under cold stress (four centigrade), the body temperature in RTM, MTM, and young groups was significantly higher than in the aging group. (j) Representative infrared image of body temperature. The color strip at the top indicated the temperature range. Color transition from blue (left) to pink (right) corresponds to temperature transition from low to high. *p < 0.05, ***p < 0.001 are considered significantly different

Next, we examined approximately 20 biochemical blood indices, which cover most of the indices indicative of the health status of organs, to screen for any positive changes. We did not find a significant difference for the mean values of most indices. However, a meticulous analysis of the distribution of values was performed with five repeats of several indices, including uric acid (UA), glutamic pyruvic aminotransferase (ALT), glutamic oxalacetic aminopherase (AST), AST/ALT, creatine kinase (CK), and glucose (Glu); this analysis revealed that although there was no statistically significant difference for the mean values between groups, the five values in the RTM, MTM, and young groups are all much closer to the average, while the five values in the aging group were scattered over a wide range and were far from each other (Figure 7a–f). These results indicate that most of the aging mice tended to have several abnormal blood indices that could affect general health.

It is known that BAT is enriched with mitochondria and is essential for thermogenesis; additionally, good thermal regulation is an essential premise for overall health (Villarroya et al., 2017, 2013; Wang et al., 2015). Next, we examined body temperature. We found that BAT xenotransplantation slightly, but significantly, elevated body temperature (Figure 7h, aging/RTM/MTM/young = 36.04:36.2:36.2:36.12). Moreover, under cold stress, the body temperature of the aging mice significantly decreased by almost 1.5 degrees centigrade compared with that of the young mice, while BAT xenotransplantation significantly elevated the body temperature to levels that were close to those of the young group (Figure 7i,j, aging/RTM/MTM/young = 34.62:35.86:35.80:35.88).

These results suggest that rat BAT xenotransplantation not only improves the fertility of aging mice but also enhances their overall health condition.

### 3.5 mRNA expression profiles in the RTM and MTM groups were partially rescued to levels close to those of the young group in both F0 and F1 mice

Next, we performed comparative mRNA sequencing to further uncover how RTM and MTM improve the overall ovarian function in both F0 and F1 model mice. We did not separate oocytes and granular cells from the ovaries for sequencing since the amount of fully grown oocytes (FGO) is very low in an ovary of a 10‐month‐old mouse (5–7 oocytes per ovary). Single‐cell sequencing is probably not possible in this case since both oocytes and granular cells within the ovary are not homogeneous. Additionally, the object we studied was the ovary as a whole, and every kind of cell (oocytes, granular cells, stromal cells, etc.) contribute to normal ovarian function, so we chose to keep the ovary intact. We can always consult the National Center for Biotechnology Information (NCBI) or Mouse Genome Informatics (MGI) databases to determine the expression pattern of a gene in various tissues and cells.

**Figure 8:**
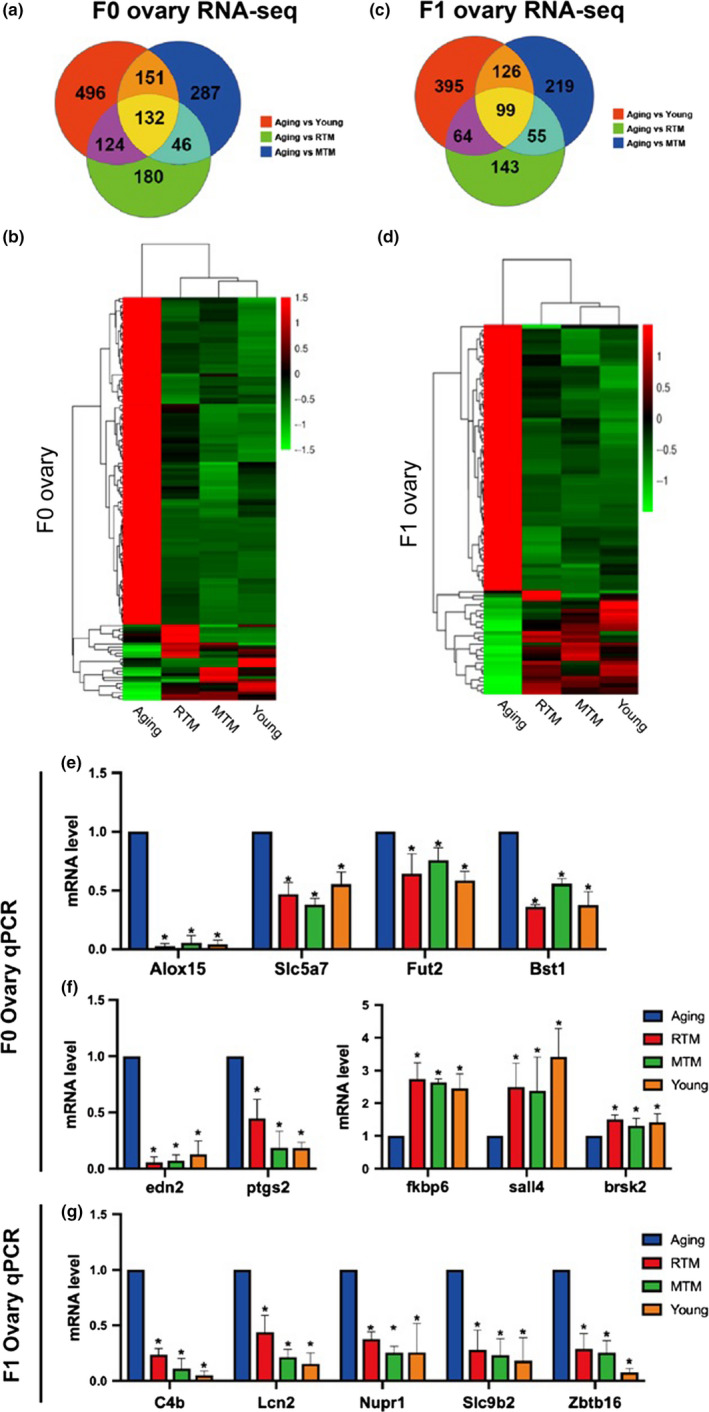
mRNA expression profiles in RTM and MTM were partially rescued close to the young group in both F0 and F1 mice. (a) Venn diagram showed that in F0 mice, compared with the aging group, there were 132 differentially expressing genes (DEGs, upregulated or downregulated) overlapped between RTM, MTM, and young groups. (b) Heat map of overlapping genes from (a). (c) Venn diagram showed that in F1 mice, compared with the aging group, there were 99 DEGs (upregulated or downregulated) overlapped between RTM, MTM, and young groups. (d) Heat map of overlapping genes from (c). (e) qPCR verified four metabolism‐related DEGs that were downregulated and overlapped between RTM, MTM, and young groups in F0 mice. (f) qPCR verified two downregulated (left) and three upregulated representative DEGs that overlapped between RTM, MTM, and young groups in F0 mice. (g) qPCR validated five representative DEGs that overlapped between RTM, MTM, and young groups in F1 mice. *p < 0.05 is considered significantly different

For both F0 (Figure 8a,b) and F1 (Figure 8c,d) mice, Venn diagrams and mRNA expression heat maps showed that, compared with the aging group, RTM, MTM, and young group had many overlapped differentially expressed genes (DEGs) (|log2| > 1, 132 for F0, 99 for F1, Dataset S3). Since the former experimental evidence showed rat BAT could rescue multiple indices indicative of follicle and oocyte quality and improve and health, we focused on the overlapping DEGs involved in follicle and oocyte quality and general health. Here, we also pay attention to metabolism‐related genes, since general health and normal metabolism are inseparably interconnected with each other.

First, KEGG pathway analysis showed that these DEGs are involved in multiple pathways essential for follicle and oocyte quality in the ovaries of both F0 and F1 (Dataset S4). We picked 20 pathways for F0 and F1 (Figure S2), and eight pathways (mTOR, PI3K‐Akt, FoxO, Toll‐like receptor, ErbB, Cytokine–cytokine receptor interaction, oocyte meiosis, apoptosis) are identical between F0 and F1 mice. Actually, 6 of 8 (mTOR, PI3K‐Akt, FoxO, Toll‐like receptor, ErbB, Cytokine–cytokine receptor interaction) are also essential for metabolism and overall health.

Second, GO classification analysis showed that these DEGs are involved in multiple biological processes (Figure S3, Dataset S5), and 17 processes are identical between F0 and F1 mice. Among these, five biological processes (cell proliferation, growth, signaling, developmental process, reproduction, metabolic process) are also correlated with follicle and oocyte quality, metabolism, and overall health.

qPCR of some upregulated and downregulated genes verified the reliability of the sequencing results (Figure 8e–g). Specifically, we confirmed that in the F0 RTM and MTM groups, the levels of multiple metabolic genes, including Alox15, Slc5a7, Fut2, and Bst1, were significantly reduced to levels that were close to the levels in the young group (Figure 8e), indicating that metabolism of F0 aging mice had been rescued in many ways.

These data indicate that rat BAT xenotransplantation could rescue not only the levels of multiple genes involved in follicle and oocyte quality but also recover the levels of various genes required for the metabolism and general health of aging mice. And signal pathways and biological processes these DEGs involved are broad and vital. Moreover, F0 and F1 mice shared multiple identical signal pathways and biological processes.

### 3.6 DNA methylation profiles in the RTM and MTM groups were partially rescued to levels close to the levels in the young group in both F0 and F1 mice

**Figure 9:**
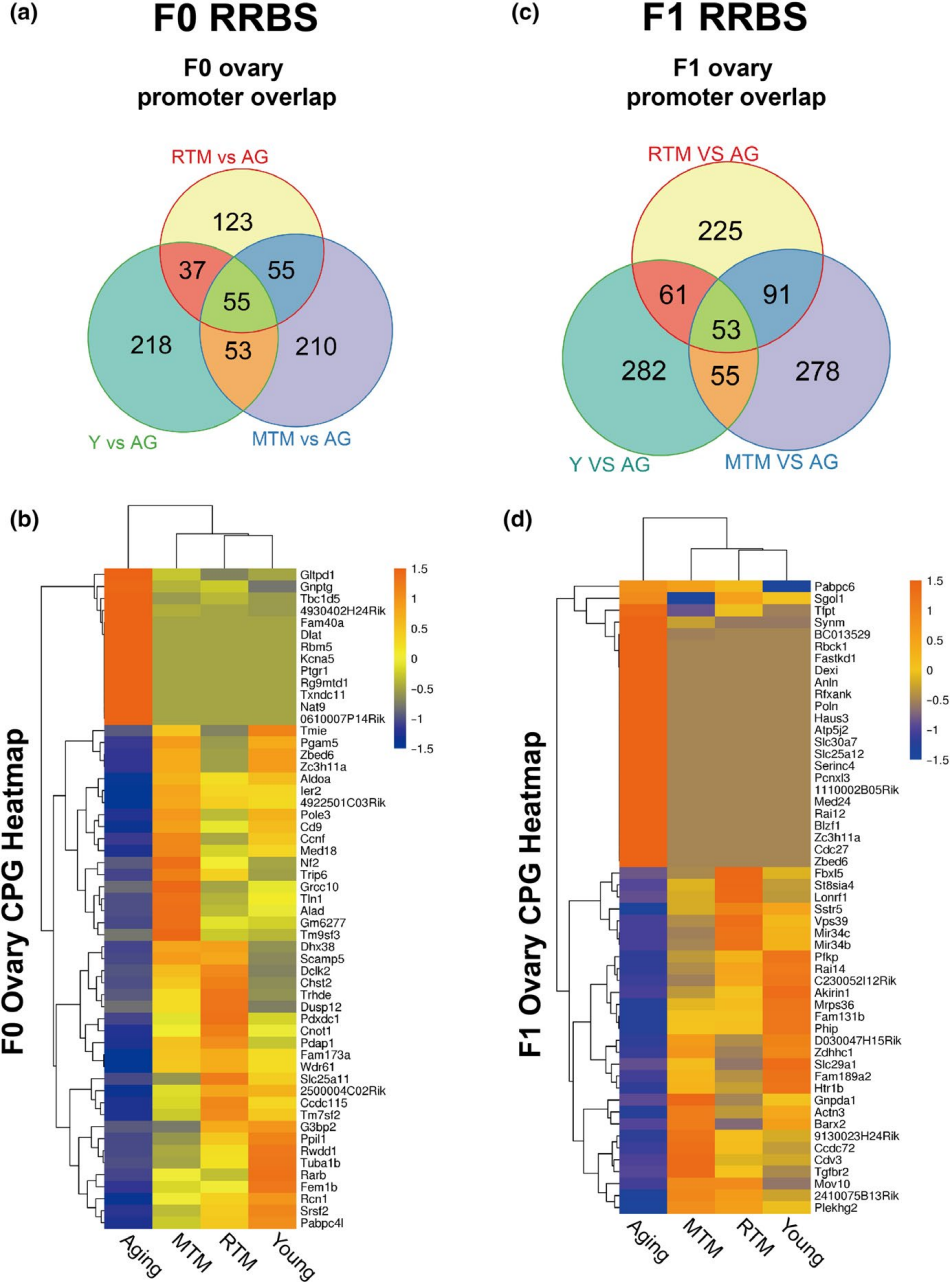
DNA methylation profiles in RTM and MTM were partially rescued close to the young group in both F0 and F1 mice. (a) Venn diagram showed that in F0 mice, compared with the aging group, there were 55 overlapped differentially methylated genes between RTM, MTM, and young groups. (b) Heat map of the methylation levels of the genes in (a) showed that there were many similarities between the MTM, RTM, and young groups in F0 mice. (c) Venn diagram showed that in F1 mice, compared with the aging group, there were 53 overlapped differentially methylated genes between RTM, MTM, and young groups. (d) Heat map of the methylation levels of the genes in (c) showed that there were many similarities between the MTM, RTM, and young groups in F1 mice

There are several studies about the effects of aging on the DNA methylation of oocytes; however, no study has been conducted to show the effects of aging on the DNA methylation of intact ovaries and whether BAT transplantation alters the methylation profiles. Therefore, we used reduced representation bisulfite sequencing (RRBS) to obtain the CpG methylation levels in different groups. Venn diagrams and heat maps of the CpG methylation levels showed that compared with those in the aging group, the levels of 55 genes in the F0 mice (Figure 9a,b, Dataset S6) and 53 genes in the F1 mice (Figure 9c,d, Dataset S6) were much closer to each other among the RTM, MTM, and young groups, indicating that the RTM and MTM treatments were able to rescue the methylation levels of many genes to levels that were close to those in the young group.

Moreover, if CHG methylation and CHH methylation were combined together with CpG methylation, then compared with those in the aging group, there were 350 (54 hypermethylated and 296 hypomethylated) differentially methylated regions (DMRs) in the F0 mice (Figure S4a) and 246 (108 hypermethylated and 138 hypomethylated) DMRs in the F1 mice (Figure S4c) overlapping among the RTM, MTM, and young groups. KEGG pathway analysis revealed that the genes associated with these DMRs are involved in multiple pathways essential for follicle and oocyte quality (Figure S4b,d, Dataset S7). Notably, multiple pathways between the F0 and F1 mice are identical (Figure S4b,d, Dataset S7), indicating that RTM and MTM function partially through a similar mechanism in the F0 and F1 mice.

### 3.7 Differential DNA methylation of the RTM versus aging groups partially overlapped between the F0 and F1 mice

**Figure 10:**
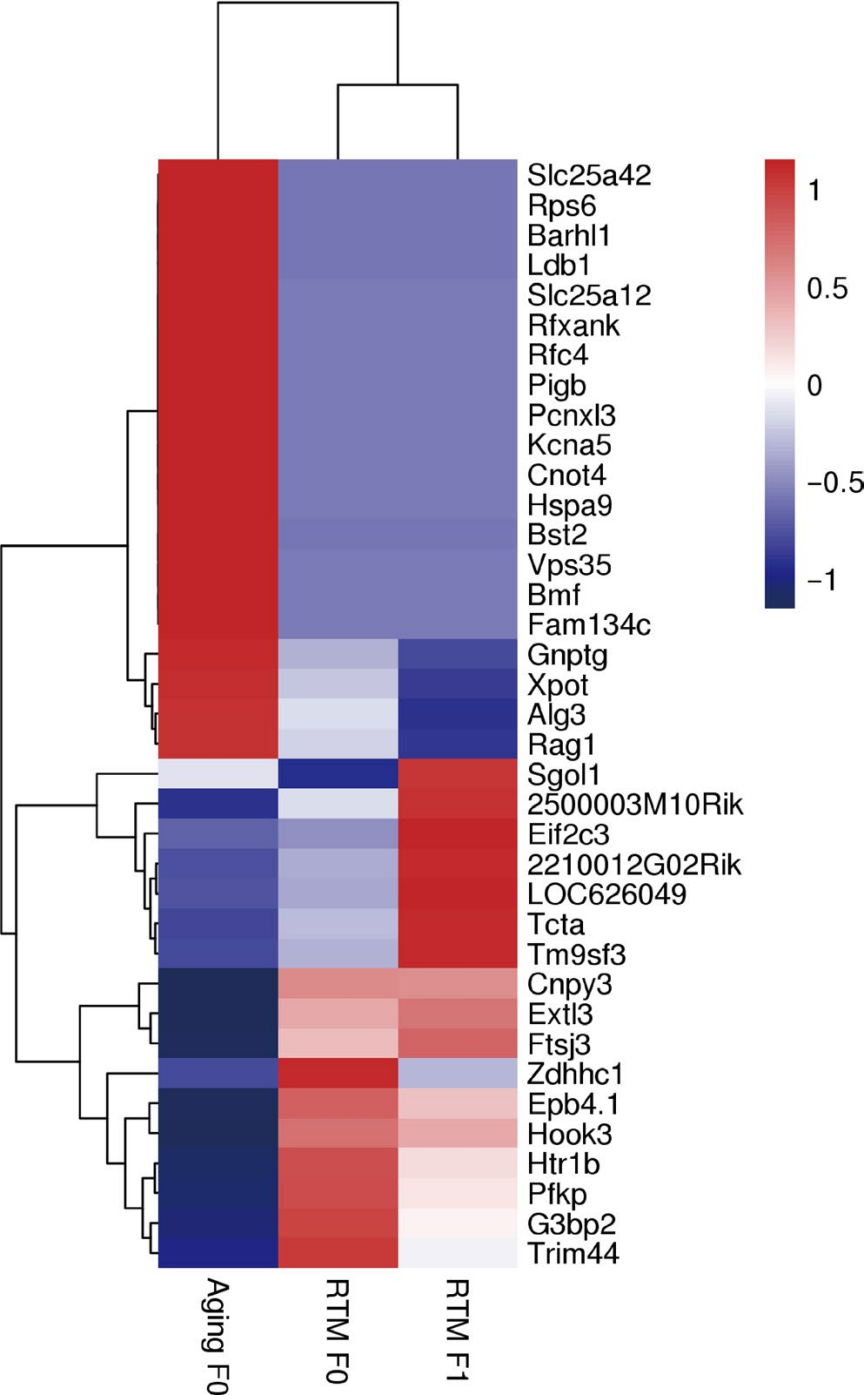
The differentially methylated genes of RTM versus aging overlapped between F0 and F1 mice. Heat map of the methylation levels showed that compared with the aging group (left), 37 differentially methylated genes overlapped between F0 RTM (middle) mice and F1 RTM (right) mice

Many recent studies have shown that epigenetic changes in the F0 generation can also cause significant changes in the F1 generation. We focused on the DMRs of the RTM versus aging groups between the F0 and F1 mice, and heat maps of the CpG methylation levels revealed that 37 genes overlapped between the F0 and F1 mice (Figure 10, Dataset S8).

Moreover, if CHG methylation and CHH methylation were combined with CpG methylation, then there were 190 (111 hypermethylated and 79 hypomethylated) overlapped DMRs in the RTM versus aging groups between the F0 and F1 mice (Figure S5a), and these DMRs corresponded to 97 genes (Figure S5b). KEGG pathway analysis revealed that these genes are involved in multiple pathways essential for follicle and oocyte quality (Figure S5c, Dataset S9), suggesting that the methylation profiles rescued by RTM in F0 mice could be passed onto F1 offspring.

Finally, string analysis showed that the proteins encoded by these epigenetically transferable genes could form an interaction net (Figure S5d).

## 4 DISCUSSION

Allotransplantation of tissues and organs frequently causes an acute rejection reaction, which is usually even worse in the case of xenotransplantation. Hence, in either animal experiments or clinical treatments, antirejection drugs must be used (Chavan, Ma, & Chiu, 2018; Morelon, Petruzzo, & Kanitakis, 2018; Sachs, 2017; Vadori & Cozzi, 2015). However, in the current study, we did not observe more obvious side effects in the RTM group than in the MTM group, as examined by measuring the expression levels of the main rejection marker HLA‐A. Previous studies have shown that allotransplantation of ADSCs mixed with inguinal adipose pads significantly increased the survival of adipose grafts because ADSCs can modulate the inflammatory and oxidative responses via Nrf2 and TLR4 (Chen et al., 2016; Zhang et al., 2015). In the present study, we did not include ADSCs; we removed and xenotransplanted only the BAT and obtained satisfying results. This might be explained by the fact that BAT contains intrinsic ADSCs that could function to reduce acute rejection, the ROS levels, and the inflammatory response as well as, or much better than extrinsically cultured ADSCs (Di Franco et al., 2014; Tran & Kahn, 2010; Wankhade & Rane, 2017). Alternatively, BATs could secrete an array of known or unknown brown adipokines (the so‐called brown adipokines or batokines), such as IL‐6, adiponectin, HGF, BFGF, fibroblast growth factor 21, and neuregulin 4, to counteract these detrimental reactions (Ding et al., 2018; Villarroya et al., 2017, 2013; Wang et al., 2015). Notably, a comparative secretome analysis has recently characterized many novel adipokines in brown adipocytes (Ali Khan et al., 2018). In the current study, we found that the levels of IL‐6 and adiponectin in the RTM and MTM groups were rescued to levels close to those in the young group. Elevated levels of serum interleukin‐6 have been reported to be associated with low‐grade cellular rejection in patients with heart transplantation (Perez‐Villa, Benito, Llancaqueo, Cuppoletti, & Roig, 2006), and it was also reported that adiponectin could inhibit allograft rejection in murine cardiac transplantation (Okamoto et al., 2009). These results provided additional support that immune rejection was trivial in our study.

Multiple studies have reported that the inhibition of the mTOR pathway could prolong the ovarian lifespan by inhibiting primordial follicle activation (Dou et al., 2017; Goldman et al., 2017; Li et al., 2015; Shay et al., 2017; Smith et al., 2013; Zhou et al., 2014). In the present study, we observed that the p‐rps6 levels significantly decreased while the p‐AMPK levels significantly increased, and consequently, the p‐MTOR levels significantly decreased. However, the number of primordial follicles tended to increase after transplantation, but this increase was not statistically significant. The reason might be that the basal reserve of the primordial follicles in aging mice is already very low (six‐ to sevenfold lower) compared with that in the young group, so the limited increase in primordial follicle count due to mTOR inhibition might not be the only (significant) cause of the significantly recovered reproduction abilities in the transplantation groups. Notably, the number of antral follicles, which could eventually lead to live birth, significantly increased by almost onefold after transplantation. At the molecular level, several key factors, such as GDF9, BMP15, LHR, and Cyp19a, which are essential for ovarian steroidogenesis, follicle development, and oocyte quality, were rescued to levels close to those of the young group. Particularly, in the RTM and MTM groups, the levels of Sirt1, a histone deacetylase whose activity increases during genotoxic, oxidative, or metabolic stress stimuli (Han et al., 2017), were rescued to levels closest to those in the young group. And moreover, ovary transcriptome study screened more DEGs required for follicle and oocyte quality and these DEGs are involved in multiple KEGG pathways (mTOR, PI3K‐Akt, FoxO, Toll‐like receptor, ErbB, Cytokine–cytokine receptor interaction, oocyte meiosis, apoptosis, etc.) and GO classified biological processes (cell proliferation, growth, signaling, developmental process, reproduction). Therefore, from these clues, we propose that the improved reproductive abilities in the RTM and MTM groups could be primarily due to the changes in multiple markers and indices indicative of the improved follicle and oocyte quality.

In addition to decreased fertility, the process of natural aging could also generally be accompanied by the functional degeneration of some other major organs and an abnormal metabolism. In our study, the xenotransplanted BAT seemed to have a positive impact on both aspects. For the first problem, as shown in the present study, the averages of the five measurements of the biochemical blood indices UA, ALT, AST, AST/ALT, CK, and Glu were much closer to each other in the RTM, MTM, and young groups than they were to those in the aging group. Uric acid reflects intrinsic purine metabolism and kidney health; ALT and AST are two primary markers for the liver; CK catalyzes the generation of phosphocreatine to support normal muscle and brain functions; and Glu (glucose) is the primary molecule that all organs can utilize directly for energy generation. The improvement of these five indices might indicate that these organs have been functionally improved. For the second problem, good thermal regulation is the premise for and is suggestive of a good overall metabolism. We found that upon cold stress, the average body temperature of the mice in the RTM and MTM groups was significantly elevated to a level close to that in the young group. Many changes could contribute to this, and we verified that UCP1, IL6, and adiponectin, which are well known to be crucial for regulating heat generation and maintaining body temperature (Villarroya et al., 2017, 2013; Wang et al., 2015; Yuan et al., 2016), were significantly upregulated in the RTM and MTM groups to levels close to those in the young group. Moreover, ovary transcriptome study screened more DEGs, and these DEGs participated in multiple KEGG pathways (mTOR, PI3K‐Akt, FoxO, Toll‐like receptor, ErbB, Cytokine–cytokine receptor interaction, pyruvate metabolism, AGE‐RAGE, NOD‐like receptor, etc.) that are essential for metabolism. In particular, multiple metabolic genes, including Alox15, Slc5a7, Fut2, and Bst1, which were significantly upregulated in the aging group, were reduced in the RTM and MTM groups close to the levels observed in the young group. Alox15 overexpression could induce osteoarthritis (Chen, Cai, et al., 2017a; Chen, Yan, et al., 2017b), tumorigenesis (Kelavkar et al., 2001), hypotension (Aggarwal et al., 2008), and anxiety (Joshi, DiMeco, & Pratico, 2014); Slc5a7 overexpression elevated acetylcholine levels and augmented motor endurance (Holmstrand et al., 2014); Fut2 overexpression also promoted cell proliferation, migration, and tumorigenicity (Lai et al., 2019) in mice. So presumably the overall metabolism might be improved when the levels of these genes were rescued.

Furthermore, comparative transcriptome‐wide studies have characterized many DEGs with essential functions other than those we have stated above. For example, among the upregulated genes, spalt‐like transcription factor 4 (Sall4) rapidly moves to DNA double‐stranded break (DSB) sites after DNA damage and is required for activating the critical ataxia telangiectasia mutated (ATM)‐dependent cellular responses to DSBs, conferring resistance to DSB‐induced cytotoxicity in mouse ESCs (Xiong et al., 2015). In oocytes, Sall4 regulates the expression of the key histone demethylase coding genes Kdm5b, Kdm6a, and Kdm6b to modulate the H3K4me3 and H3K27me3 modifications that are critical for oocyte maturation and meiosis resumption (Xu et al., 2017). Fkbp6 has been shown to be a component of the synaptonemal complex and is essential for homologous chromosome pairing (Crackower et al., 2003; Noguchi et al., 2008) and the phosphorylation of H2AX (Noguchi et al., 2008) in male meiosis. As an example of the downregulated genes, prostaglandin‐endoperoxide synthase 2 (Ptgs2), also known as Cox2, is important for ovulation (Hester, Harper, & Duffy, 2010). However, Cox2 overexpression can also induce tumorigenesis (Chen, Cai, et al., 2017a; Chen, Yan, et al., 2017b).

Many nongenetic factors can induce broad changes in DNA methylation, which subsequently modulate the transcriptional levels of various genes, eventually affecting diverse biochemical processes. It has been reported that aging could significantly alter DNA methylation, which is closely correlated with aging‐associated tumorigenesis and degenerative diseases (Field et al., 2018; Klutstein, Nejman, Greenfield, & Cedar, 2016; Sen, Shah, Nativio, & Berger, 2016). To the best of our knowledge, no epitome‐wide studies are available about the question of whether aging affects DNA methylation in intact ovaries in aging mice. Our studies show for the first time that aging significantly affects DNA methylation in the intact ovaries of aging mice and that BAT transplantation could largely rescue the methylation profiles of many genes.

Furthermore, differences in DNA methylation profiles between the RTM and aging groups show considerable overlap between the F0 and F1 mice. One explanation is that the methylation change in F0 RTM oocytes could be passed onto F1 mice. Recently, it was reported that paternally aging‐associated DNA methylation changes in sperm could affect the methylation profiles of tissues in the next generation (Xie et al., 2018). Another explanation could be that the overlap was not due to causality but rather represented a common phenomenon that improved the health of the F0 RTM mother and will significantly benefit the fetus during pregnancy. Currently, we cannot provide more evidence for any of these explanations. Nevertheless, for the first time, our study suggests that the DNA methylation of many genes in the ovaries can be broadly affected by aging and that RTM could rescue the methylation profiles to be close to those in young mice; F1 mouse methylation profiles showed much overlap with the F0 mother.

In conclusion, for the first time, we have proven that a single RTM BAT xenotransplantation could significantly improve the fertility and global health of aging mice. The cause is likely a combination of improved quality antral follicles and oocytes. Rat‐to‐mouse improved the transcriptional and DNA methylation profiles in F0 and F1 mice. This study could be a helpful reference for clinical BAT xenotransplantation from close human relatives (such as gorillas and monkeys, Figure S6) to woman.

## CONFLICT OF INTEREST

The authors declared that they have no conflicts of interest to this work.

## AUTHOR'S CONTRIBUTION

Dong Zhang, Qing‐Yuan Sun, and Fu‐Qiang Wang designed the research with the assistance of Zhi‐Xia Yang. Zhi‐Xia Yang also coordinated between authors for the experiment assignment. Liang‐Jian Chen, Yang Wang, Lei Du, Yan‐Ru Li, Na‐Na Zhang and Wen‐Yi Gao performed most of the experiments, data collection and analysis; all the others assisted them during the whole processes. Liang‐Jian Chen also did most of the figure preparation under the supervision of Dong Zhang, Qing‐Yuan Sun, and Fu‐Qiang Wang. Dong Zhang wrote the manuscript with the assistance of Liang‐Jian Chen and Zhi‐Xia Yang; Qing‐Yuan Sun and Fu‐Qiang Wang proofread and gave advice. All authors read and approved the final manuscript.
