# Supplementary figures and images for: Single xenotransplant of rat brown adipose tissue prolonged the ovarian lifespan of aging mice by improving follicle survival
Source: Aging Cell. 2019 Aug 6;18(6):e13024. doi: 10.1111/acel.13024 (PMC6826128; doi:10.1111/acel.13024)

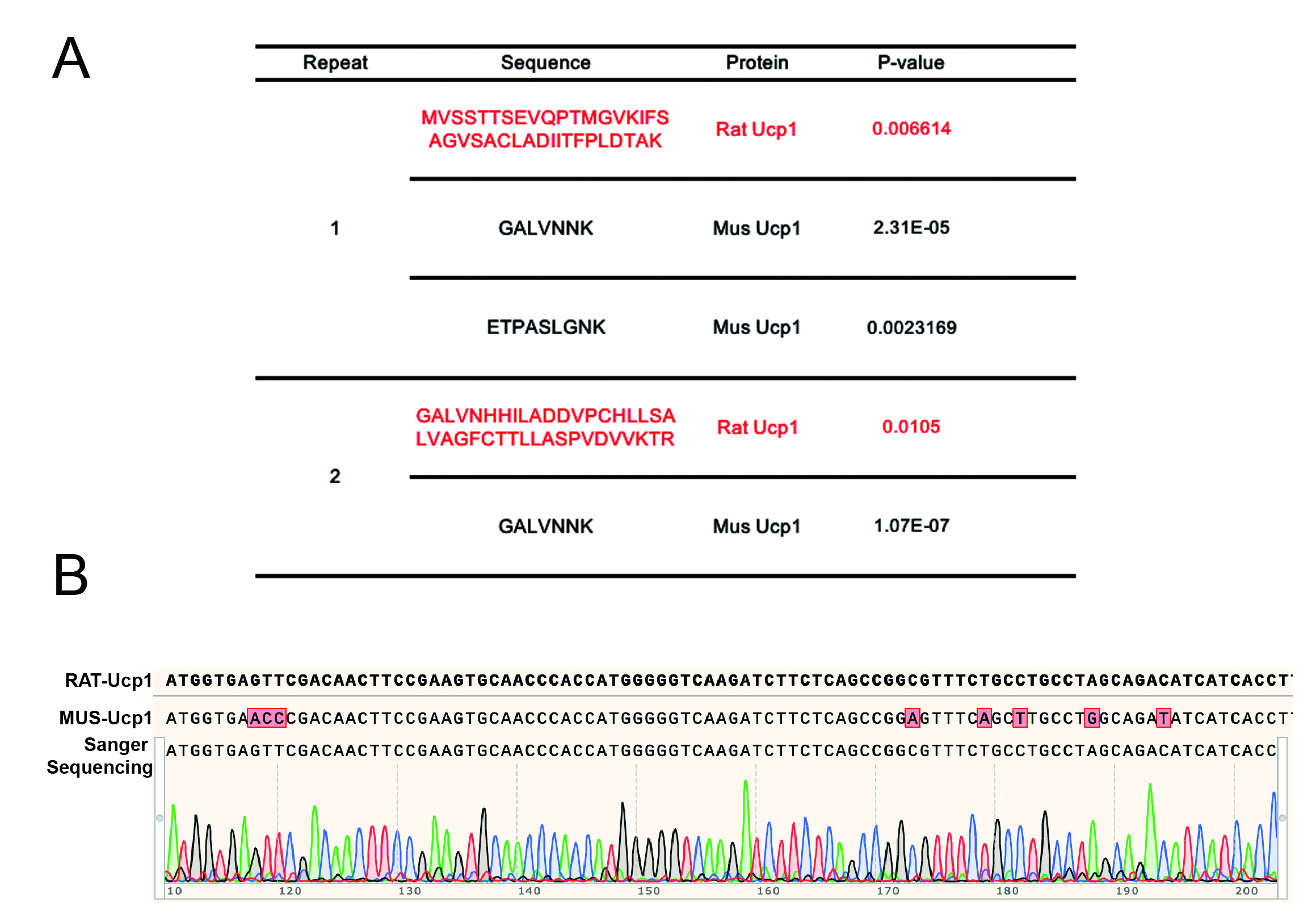

Supplement: Supplementary file 1 [file ACEL-18-e13024-s005.jpg]

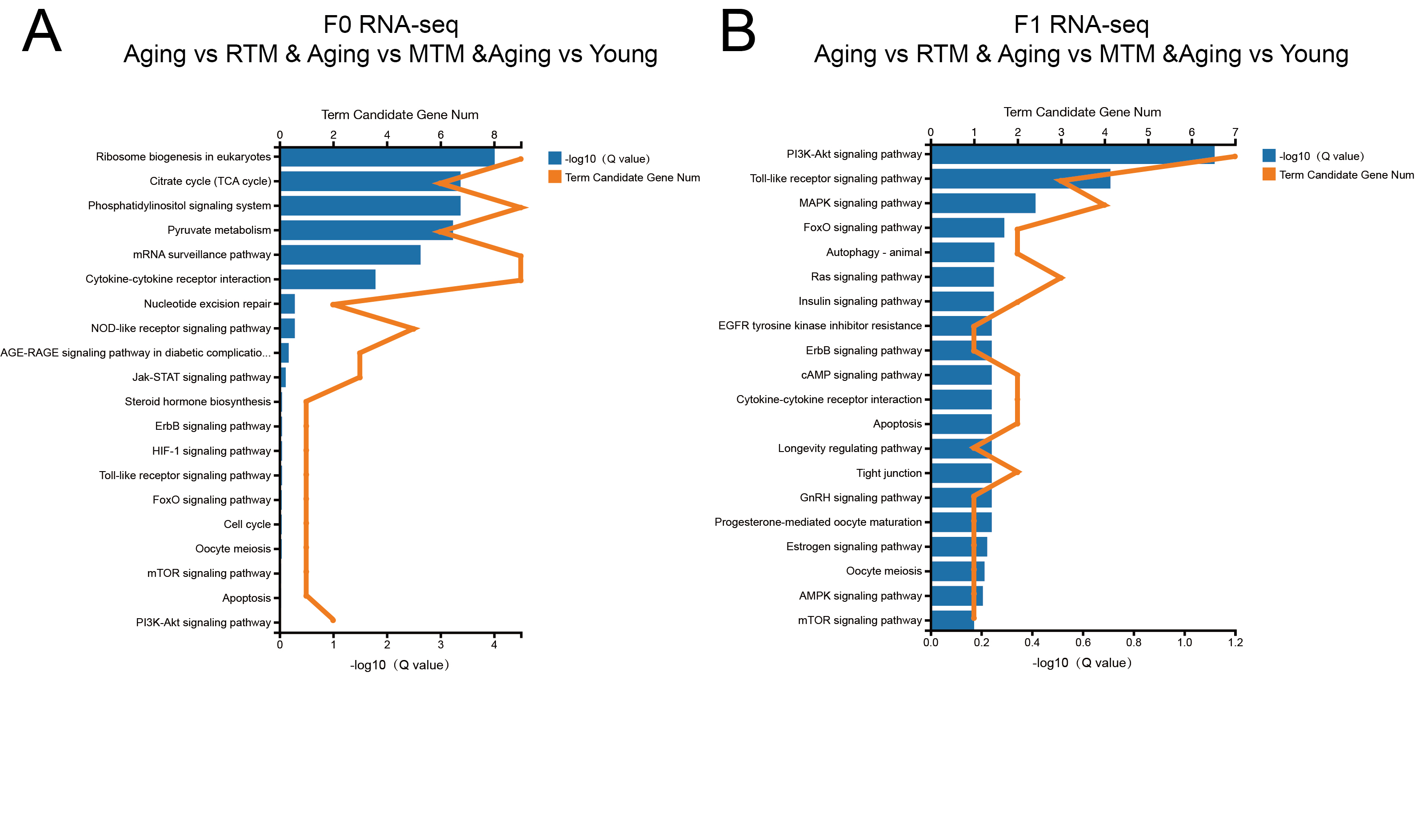

Supplement: Supplementary file 2 [file ACEL-18-e13024-s004.jpg]

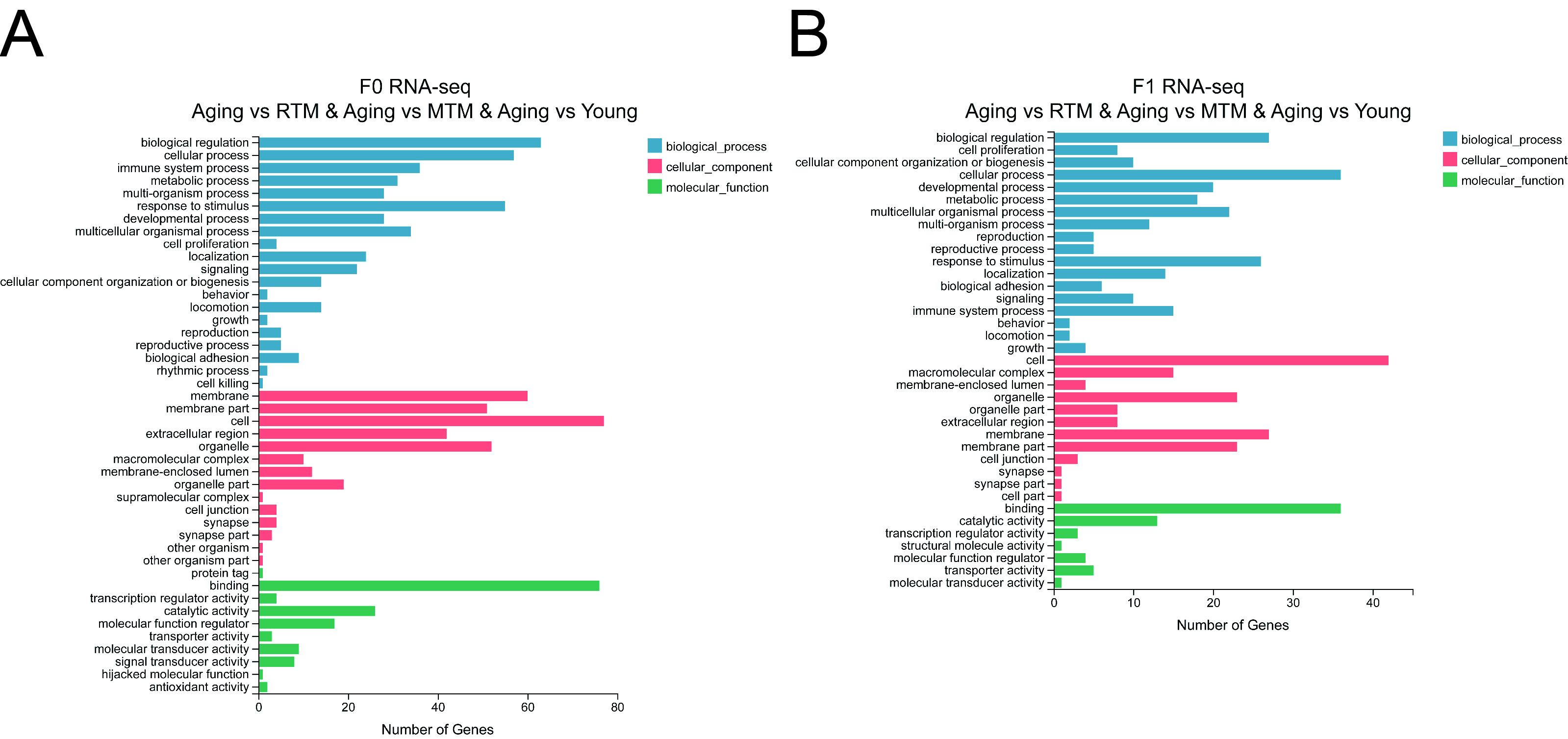

Supplement: Supplementary file 3 [file ACEL-18-e13024-s006.jpg]

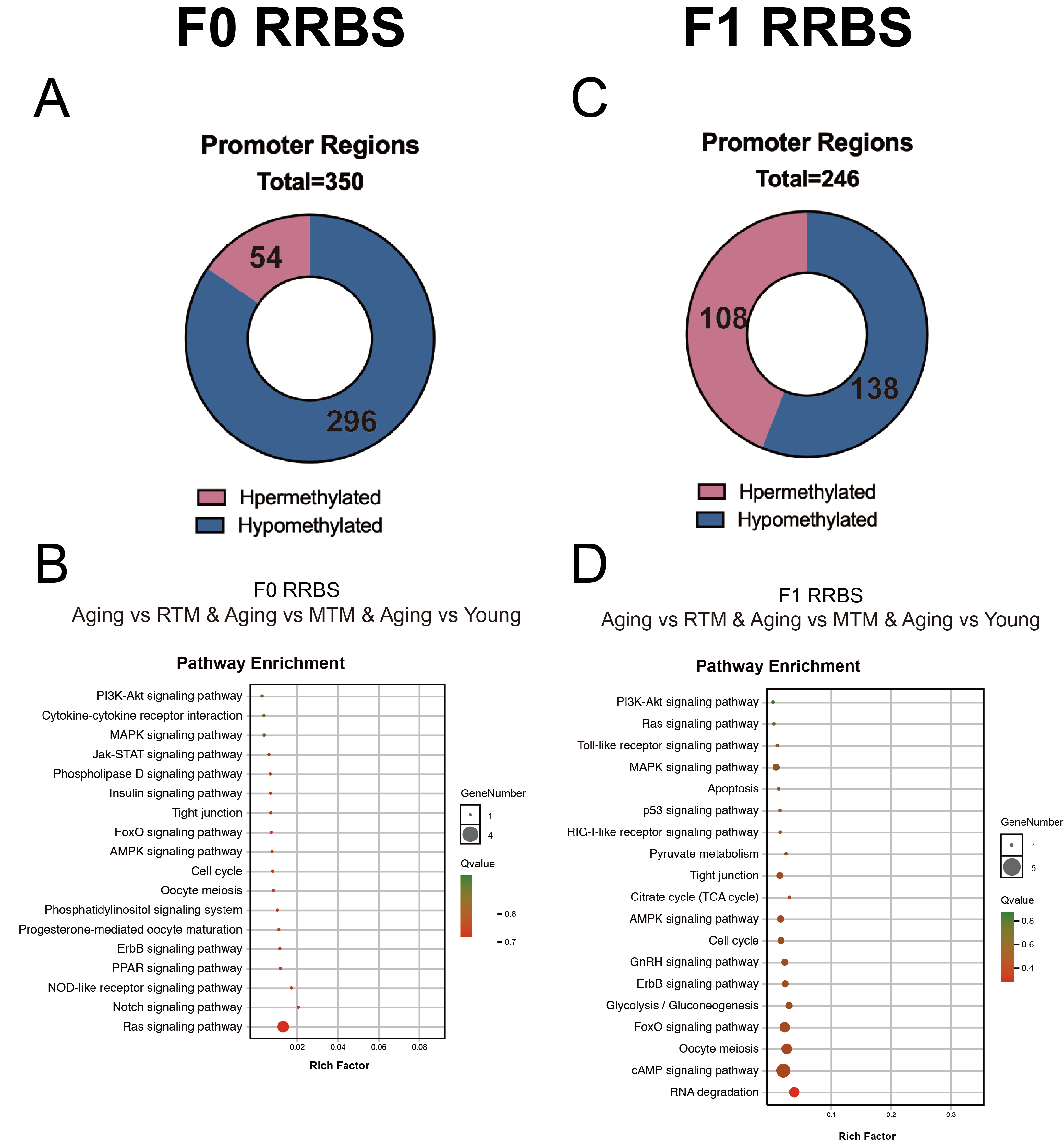

Supplement: Supplementary file 4 [file ACEL-18-e13024-s007.jpg]

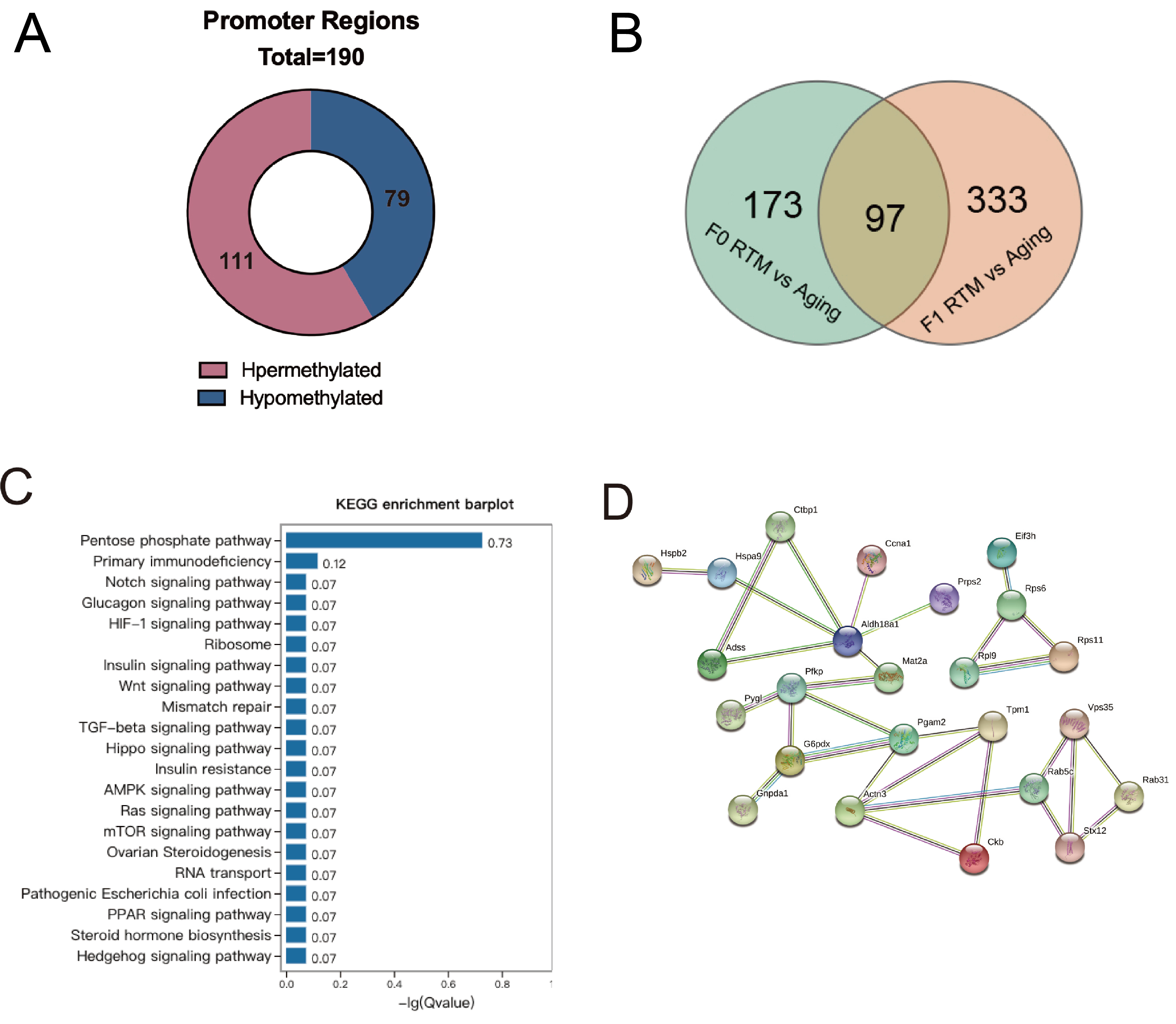

Supplement: Supplementary file 5 [file ACEL-18-e13024-s001.jpg]

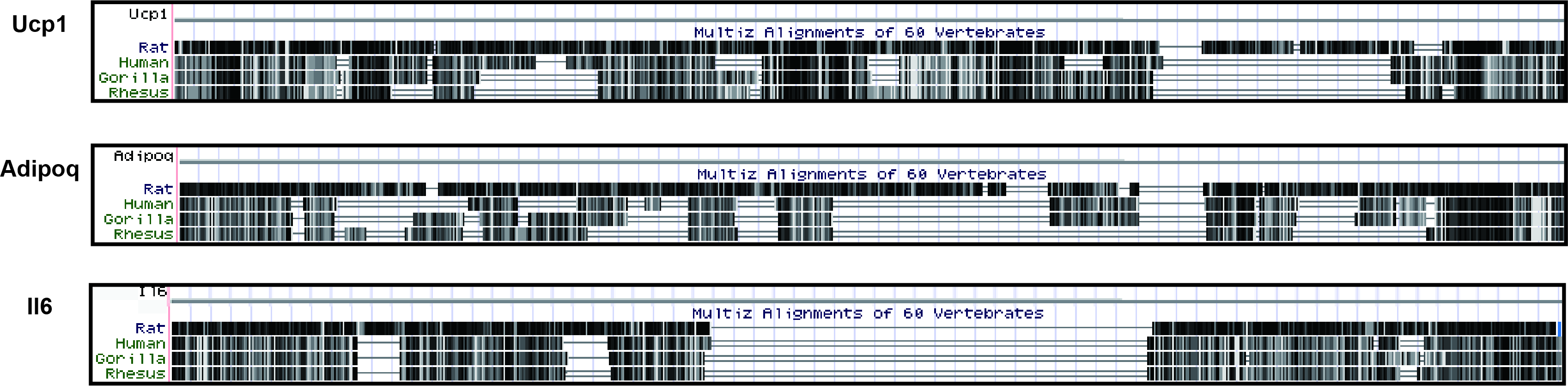

Supplement: Supplementary file 6 [file ACEL-18-e13024-s002.jpg]
