# Supplementary material for: Single xenotransplant of rat brown adipose tissue prolonged the ovarian lifespan of aging mice by improving follicle survival
Source: Aging Cell. 2019 Aug 6;18(6):e13024. doi: 10.1111/acel.13024 (PMC6826128; doi:10.1111/acel.13024)
Supplement: Supplementary file 7 [file ACEL-18-e13024-s003.docx]

| Gene ID | | Sequence |
| --- | --- | --- |
| Rat-ucp1^1^ | Forward | CACCTTTGAGCTCCTCCAC |
|  | Reverse | GGTGATGATGTCTGCTAGGC |
| Ucp1^1,2^ | Forward | TGCGCCTTCTTTTCTGCGAC |
|  | Reverse | CCGGGACTTCATCAGCTCTTTC |
| PPARα^2^ | Forward | GCTTCTTTCGGCGAACTAT |
|  | Reverse | TTTCCTGCGAGTATGACCC |
| PGC1α^2^ | Forward | ATGTGTCGCCTTCTTGCTCT |
|  | Reverse | ATCTACTGCCTGGGGACCTT |
| MCAD^2^ | Forward | AGGTGTTTGGTGAGATAGTCG |
|  | Reverse | ATCCATGGCTTCGTACTTGC |
| GDF9^3^ | Forward | GGCATATGGGTGTACAGGGG |
|  | Reverse | ACGCAGTAGGCACACATCAT |
| BMP15^3^ | Forward | TCCTTGCTGACGACCCTACAT |
|  | Reverse | TACCTCAGGGGATAGCCTTGG |
| LHr^3^ | Forward | CGCCCGACTATCTCTCACCTA |
|  | Reverse | GACAGATTGAGGAGGTTGTCAAA |
| Sirt1^3^ | Forward | GCTGACGACTTCGACGACG |
|  | Reverse | TCGGTCAACAGGAGGTTGTCT |
| Cyp19α^3^ | Forward | ATGTTCTTGGAAATGCTGAACCC |
|  | Reverse | AGGACCTGGTATTGAAGACGAG |
| Fkbp6^4^ | Forward | CGCCTCAGGAACGGAATCC |
|  | Reverse | TGACTTAGTCGCTCATAGGGAG |
| Sall4^4^ | Forward | CCCTGGGAACTGCGATGAAG |
|  | Reverse | TCAGAGAGACTAAAGAACTCGGC |
| Brsk2^4^ | Forward | ACCTGCTGCTAGATGAGAGGA |
|  | Reverse | CTCGCCCCGAATCACTTCC |
| Edn2^4^ | Forward | CACCTGCGTTTTCGTCGATG |
|  | Reverse | CCAGTGTCTTCGATGGCAGAA |
| Ptgs2^4^ | Forward | TGAGCAACTATTCCAAACCAGC |
|  | Reverse | GCACGTAGTCTTCGATCACTATC |
| Alox15^5^ | Forward | GGCTCCAACAACGAGGTCTAC |
|  | Reverse | AGGTATTCTGACACATCCACCTT |
| Slc5a7^5^ | Forward | ATGTCTTTCCACGTAGAAGGACT |
|  | Reverse | TTGCCGCTGTTTTTGGTTTTC |
| Fut2^5^ | Forward | ACCTCCAGCAACGAATAGTGA |
|  | Reverse | GCCGATGGAATTGATCGTGAA |
| Bst1^5^ | Forward | AGGGACAAGTCACTGTTCTGG |
|  | Reverse | AACTTTGCCATACAGCACGTC |
| C4b^5^ | Forward | ACTTCAGCAGCTTAGTCAGGG |
|  | Reverse | GTCCTTTGTTTCAGGGGACAG |
| Lcn2^5^ | Forward | TGGCCCTGAGTGTCATGTG |
|  | Reverse | CTCTTGTAGCTCATAGATGGTGC |
| Nupr1^5^ | Forward | CCCTTCCCAGCAACCTCTAAA |
|  | Reverse | TCTTGGTCCGACCTTTCCGA |
| Slc9b2^5^ | Forward | GCGAGCCTTTCTGGTTCTG |
|  | Reverse | CACCTCATGCCTGCTAGGA |
| Zbtb16^5^ | Forward | CTGGGACTTTGTGCGATGTG |
|  | Reverse | CGGTGGAAGAGGATCTCAAACA |
| Actin | Forward | CCGTAAAGACCTCTATGCC |
|  | Reverse | CTCAGTAACAGTCCGCCTA |

^1^These 2 primers are used for Figure 1C

^2^These 4 primers are used for Figure 1F

^3^These 5 primers are used for Figure 5D

^4^These 9 primers are used for Figure 7E

^5^These 5 primers are used for Figure 7F
