# Supplementary material for: Single xenotransplant of rat brown adipose tissue prolonged the ovarian lifespan of aging mice by improving follicle survival
Source: Aging Cell. 2019 Aug 6;18(6):e13024. doi: 10.1111/acel.13024 (PMC6826128; doi:10.1111/acel.13024)
Supplement: Supplementary file 8 [file ACEL-18-e13024-s008.docx]

**Supplementary dataset legends**

**Supplementary dataset S1.**

This file outlines the schedule that each male mouse rotates among different mating cages (1 female mouse per cage).

**Supplementary dataset S2.**

This file is for UCP1 immunoprecipitation and LC-MS with rat BAT after 6 months of xenotransplantation. UCP1 protein identification was based on the peptide identification and sequence blast search in the protein database. Please notice that the file only outlines UCP1-related original identification data. It contains four sheets. "Repeat 1 Protein identification" outlines the original UCP1 protein identification data from repeat 1; "Repeat 1 Peptide identification" outlines the original UCP1 peptide identification data from repeat 1; "Repeat 2 Protein identification" outlines the original UCP1 protein identification data from repeat 2; "Repeat 2 Peptide identification" outlines the original UCP1 peptide identification data from repeat 2. Please notice that due to the high similarity of rat UCP1 and mouse UCP1 protein, some identified peptides are unique for rat UCP1 or mouse UCP1, while the others are the same for rat UCP1 or mouse UCP1.

**Supplementary dataset S3.**

This file contains two sheets. "F0 RNA-seq overlap gene" corresponds to Figure 7B and contains all 132 F0 overlapped differentially expressed genes between RTM, MTM, and Young group compared with the Aging group and the average FPKM. "F1 RNA-seq overlap gene" corresponds to Figure 7D and contains all 99 F1 overlapped differentially expressed genes and average FPKM.

**Supplementary dataset S4.**

This file contains two sheets. "F0 RNA-seq KEGG" corresponds to supplementary figure 2A and contains all 132 F0 overlapped differentially expressed genes between RTM, MTM, and Young group compared with the Aging group and related pathways. "F1 RNA-seq overlap gene" corresponds to supplementary Figure 2B and contains all 99 F1 overlapped differentially expressed genes and related pathways.

**Supplementary dataset S5.**

This file contains two sheets. "F0 RNA-seq GO" corresponds to supplementary Figure 3A and contains all 132 F0 overlapped differentially expressed genes between RTM, MTM, and Young group compared with the Aging group and GO classification. "F1 RNA-seq overlap gene" corresponds to supplementary Figure 3B and contains all 99 F1 overlapped differentially expressed genes and GO classification.

**Supplementary dataset S6.**

This file contains two sheets. " F0 Overlap CPG" corresponds to Figure 9B and contains all 55 differentially methylated genes between RTM, MTM, and Young group compared with the Aging group and absolute methylation level (%). "F1 Overlap CPG" corresponds to Figure 9D and contains all 53 differentially methylated genes and absolute methylation level (%).

**Supplementary dataset S7.**

This file contains two sheets. In "F0 DMR KEGG", F0 KEGG enrichment analysis of 350 (54 hypermethylated, 296 hypomethylated) overlapped DMRs (supplementary Figure 4A) showed that the genes these DMRs belong to are involved in multiple essential pathways essential for follicle & oocyte quality (supplementary Figure 4B). **B.** In "F1 DMR KEGG", F1 KEGG enrichment analysis of 246 (108 hypermethylated, 138 hypomethylated) (supplementary Figure 4C) overlapped DMRs showed that the genes these DMRs belong to are involved in multiple essential pathways essential for follicle & oocyte quality (supplementary Figure 4D). Notably, multiple pathways between F0 and F1 are identical.

**Supplementary dataset S8.**

This file corresponds to Figure 9 and contains all 37 overlapped differentially methylated genes of RTM vs. Aging between F0 and F1 mice and absolute methylation level (%).

**Supplementary dataset S9.**

This file corresponds to supplementary Figure 4C and contains KEGG enrichment analysis of all 97 overlapped differentially methylated genes of RTM vs. Aging between F0 and F1 mice. It showed that these genes are involved in multiple pathways essential for follicle & oocyte quality.
